# Stress-induced enrichment of *Pseudomonas* sp. stimulates the adaptive response of *Auxenochlorella pyrenoidosa* and antibiotic-resistant proliferation

**DOI:** 10.1186/s40168-026-02335-7

**Published:** 2026-02-23

**Authors:** Qian Liu, Jia Jia, Xin Chen, Chenxi Wu

**Affiliations:** 1 Aquatic Biodiversity and Water Ecological Environment Protection Research Center, Institute of Hydrobiology, Chinese Academy of Sciences, Wuhan, 430072 China; 2 Nanchang Normal University, Nanchang, 330032 China; 3 Institute of Chemistry, Hubei Institute of Measurement and Testing Technology, Wuhan, 430073 China; 4 Qinghai Lake Comprehensive Observation and Research Station, Chinese Academy of Sciences, Haibei, 812200 China

**Keywords:** Antibiotic, Microplastic, Phycosphere, Phycospheric bacteria, Antibiotic resistance gene

## Abstract

**Background:**

The phycosphere is an important ecological niche for bacteria and antibiotic resistance genes (ARGs). However, whether and how the interaction between microalgae and bacteria changed, and its further effect on the transmission of ARGs under pollutant stress remains enigmatic. Here, *Auxenochlorella pyrenoidosa* was co-cultured with bacteria screened from lake water to explore the algal–bacteria interaction and ARGs’ transmission in the presence of florfenicol (FF) and polylactic acid microplastics (PLA MPs).

**Results:**

Our study demonstrated that the growth and metabolism of *A. pyrenoidosa* were promoted under FF treatment or co-treatment with PLA MPs, validated by phenotypic, transcriptome, and metabolome analyses. In contrast, the abundance of phycospheric bacteria was decreased as a result of niche competition. Nonetheless, the transmission of ARGs in the phycosphere was promoted due to the enrichment of antibiotic-resistant bacteria, especially *Pseudomonas*, rather than horizontal gene transfer. The algal-bacteria co-culture experiment further suggested that vitamin B6 secreted by *Pseudomonas* sp. likely contributes to underpinning *A. pyrenoidosa’* survival under FF and PLA MPs stress.

**Conclusions:**

These findings underscore the dynamic interplay and co-evolution between algae and bacteria under pollutant exposure, and reveal a potential mechanism of vitamin B6-mediated mutualism. This study provides new insights into the assembly of phycospheric bacterial communities and the adaptive strategies of microalgae in contaminated aquatic environments.

Video Abstract

**Supplementary Information:**

The online version contains supplementary material available at 10.1186/s40168-026-02335-7.

## Introduction

As primary producers in aquatic systems, microalgae play important roles in ecosystem stability and health [1]. However, due to their relatively undifferentiated cellular organization as well as sensitive physiological properties and biological functions, microalgae are susceptible to environmental pollutants [2]. The continuous release of antibiotics, which are widely used in the prevention and control of human and aquaculture diseases, not only has a serious negative impact on the physiological and metabolic activities of microalgae [3–5] but also causes the proliferation and spread of antibiotic-resistant bacteria [6, 7]. Meanwhile, as an emerging pollutant, microplastics have attracted much attention in recent years, and their impacts on aquatic environments are being continuously discovered [8, 9]. In addition to their effects on microalgal growth, microplastics can also act as enrichment vectors for antibiotics and antibiotic resistance genes (ARGs), resulting in combined toxic effects [10, 11]. Therefore, microalgae will inevitably face the dual challenges of antibiotics and microplastics.

Recent studies found that antibiotic exposure within a concentration range could promote algal growth [12, 13], indicating the potential of algae to adapt to stress. Besides, the host-associated microbiome, also known as the second genome of hosts, has an important impact on host adaptation [14–16]. In our previous research, it was also found that the addition of exogenous bacteria promoted the growth of *Chlorella pyrenoidosa* [17]. In natural water, microalgae release various metabolites to their surroundings, which constitute a distinct niche for heterotrophic bacterial colonization, called phycosphere [18, 19]. In the phycosphere, microalgae could recruit specific bacteria based on their own needs. For example, B12-dependent strains of *Chlamydomonas reinhardtii* tend to recruit bacteria that release more B12, resulting in a mutualistic relationship [20, 21]. Besides, microalgae can use their secreted metabolites to actively regulate their associated microbial communities. Shibl et al. (2020) found that the ubiquitous diatom *Asterionellopsis glacialis* utilized azelaic acid and rosmarinic acid to selectively promote the growth and attachment of symbiotic bacteria while simultaneously suppressing the colonization of other opportunistic bacteria [22]. Thus, microalgal secretions shape the activity and diversity of phycospheric bacteria, and once microalgal metabolism is altered by various abiotic stresses, the recruitment of phycospheric bacteria can also be affected [23–25]. Meanwhile, these bacteria may also regulate the growth of microalgae through chemical mediators [26, 27]. Some bacteria can provide essential nutrients for microalgae [20], while others may release algaecides to kill microalgae and then use the released substances for their own growth [28]. A previous study also found that bacteria could regulate the methionine cycle and trigger vesicle production in microalgae, thereby transporting harmful metabolites out of the microalgae and enabling their re-proliferation [29]. These results indicate that there is a close, bidirectionally regulated symbiotic relationship between microalgae and phycospheric bacteria, which can dynamically cope with environmental changes. However, the specific adaptation mechanisms of this symbiotic system in response to pollutant stress remain to be further clarified.

Meanwhile, it is worth noting that changes in bacterial communities are a key driver of ARGs’ composition in natural waters [30, 31]. Recent studies reveal that the phycosphere is also an important reservoir of ARGs and plays a non-negligible role in ARG transmission in aquatic environments [5, 6, 17]. On the one hand, phycospheric bacteria can be under the direct selection pressure of antibiotics like free-living bacteria, leading to the development of antibiotic resistance [6, 30, 32]. On the other hand, pollutant-mediated changes in microalgal metabolism may indirectly reshape the ARG composition in the phycosphere by disturbing the recruitment of antibiotic-resistant bacteria [5]. However, most current understanding, including from our previous work, has been derived from studies focusing on the exponential growth phase of microalgae [17]. The assembly of the phycosphere microbiome and its functional outcomes might differ significantly during the stationary phase, as algal metabolic activity and exudate profiles shift substantially [33]. Therefore, the occurrence of antibiotic-resistant bacteria and the antibiotic resistome driven by microalgal adaptation mechanisms under pollutant stress deserves further attention.

To bridge this knowledge gap, this study employed a long-term co-culture system to elucidate the adaptive mechanisms of algae-bacteria symbionts and the proliferation of ARGs under pollutant stress in a more stable ecosystem. In general, *Auxenochlorella pyrenoidosa* was co-cultured with natural microbial communities under the exposure of exogenous pollutants, florfenicol (FF) and polylactic acid microplastics (PLA MPs), to explore the response mechanism of algae-bacterial symbionts to pollutant stress. Multi-omics analysis revealed that phycospheric bacteria played a key role in enhancing the adaptation of microalgae to pollutant stress and the development of antibiotic resistance. Thus, we isolated the phycospheric bacteria to further validate this result. The genome of bacterial isolates was first sequenced to provide deeper insights into the mechanisms and bacterial features associated with growth-promoting effects. Then, the interrelationships among them were verified by implementing co-cultivation experiments with axenic *A. pyrenoidosa*.

## Materials and methods

### Microalgae strain and co-culture conditions

The axenic *A. pyrenoidosa* was obtained from the Institute of Hydrobiology, Chinese Academy of Sciences, Wuhan, China. The bacteria were isolated from water samples collected from South Lake (114°20′29.15″E, 30°29′37.73″N, Wuhan, China). The specific separation method was described in our previous study [17]. The obtained bacteria and axenic *A. pyrenoidosa* were co-inoculated into conical flasks containing 800 mL BG-11 medium at an initial density of 5.0 × 10^4^ ind/mL for *A. pyrenoidosa* and 1.0 × 10^6^ CFU/mL for bacteria. The flasks were incubated for 60 days at 25 ± 1 °C, illuminated with 3000 ± 300 lx with a 12 h/12 h light/dark interval, and shaken at least three times daily.

### Exposure experiments

In the exposure experiment, seven groups were included as follows: C (axenic *A. pyrenoidosa*), CB (axenic *A. pyrenoidosa* + bacteria), P (axenic *A. pyrenoidosa* + bacteria + 10 mg/L PLA MPs), FL (axenic *A. pyrenoidosa* + bacteria + 0.1 mg/L FF), PFL (axenic *A. pyrenoidosa* + bacteria + 0.1 mg/L FF + 10 mg/L PLA MPs), FH (axenic *A. pyrenoidosa* + bacteria + 10 mg/L FF), PFH (axenic *A. pyrenoidosa* + bacteria + 10 mg/L FF + 10 mg/L PLA MPs). Each treatment was performed in triplicate. The concentrations of PLA MPs and FF mentioned above were chosen with full reference to previous studies and environmentally relevant levels. The PLA concentration (10 mg/L) reflected estimated environmental mass concentrations, while the FF levels encompassed both an environmentally relevant concentration (0.1 mg/L) and an extreme polluted concentration (10 mg/L) to explore community succession under strong selection pressure [34–36]. Since no significant degradation of FF in the culture system was observed in the pre-experiment (see Text S1 and Table S1 for details), no additional FF was added to the culture system during the incubation period. The physiological responses of *A. pyrenoidosa* to FF and PLA MPs were revealed by the measurement of algal density, photosynthetic pigments, extracellular polymeric substances (EPS), and antioxidant capacity, as well as morphological observation (see Text S2 for details).

### Transcriptomic analysis

After 60-day incubation, *A. pyrenoidosa* cells were separated from the suspensions through sterile filters with a 3-µm pore size. Total RNA was extracted using TRIzol® Reagent. Transcriptome sequencing was performed via the Illumina Novaseq 6000 platform (Illumina, USA). More details on sequencing, identification of differentially expressed genes (DEGs), and functional enrichment are provided in Text S3. The sequence raw data have been deposited in the NCBI Sequence Read Archive database under the accession number PRJNA1171622.

### Non-target metabolomic analysis

Ultra-high-performance liquid chromatography-mass spectrometry (UHPLC-MS, Thermo Fisher, USA) was utilized for metabolomic analysis of *A. pyrenoidosa* in an untargeted manner. More details on metabolite extraction and determination, as well as differential accumulated metabolites (DAMs) analysis, are provided in Text S4. The raw data of the metabolome have been deposited in the MetaboLights database under the accession number MTBLS11371.

### Bacterial sampling and metagenomic analysis

At the end of incubation, the microalgal suspension was passed through a sterile filter with a pore size of 3 µm to obtain phycospheric (PS) bacteria, and the filtrate was further passed through a sterile filter with a pore size of 0.22 µm to obtain free-living (FL) bacteria. Total DNA was extracted using the E.Z.N.A. Soil DNA kit (Omega Bio-Tek, USA) according to the instructions. The DNA extract was then used for microbial sequencing and determination of ARGs. More details on sequencing, taxonomic and functional annotation, and determination of ARGs and mobile genetic elements (MGEs) are provided in Text S5–S8. The sequence raw data have been deposited in the NCBI Sequence Read Archive database under the accession number PRJNA1027504.

### Isolation of phycospheric bacteria and whole genome sequencing

Phycospheric bacteria were isolated from the PFH group after co-cultivation using a dilution-to-extinction approach (see Text S9 for details). Identification of bacterial isolates and their potential functional analysis were performed by whole genome sequencing (see Text S10 for details). The sequence raw data have been deposited in the NCBI Sequence Read Archive database under the accession numbers PRJNA1171391 (*Pseudomonas*_sp1), PRJNA1171480 (*Methylobacterium*_sp1), and PRJNA1171486 (*Allorhizobium*_sp1), respectively.

### The co-culture experiment of phycospheric isolates and A. pyrenoidosa

Based on the minimum inhibitory concentration (MIC) test (Text S11), a more dominant strain of phycospheric bacteria was selected for co-culture with axenic *A. pyrenoidosa*. Different FF and PLA MPs treatments were set up in the co-culture system, and *A. pyrenoidosa* without bacterial inoculum served as the control (see Text S12 for details). The cell density of *A. pyrenoidosa* was measured every 48 h. Algal suspensions were collected every 4 days for quantification of chlorophyll, EPS, antioxidant capacity, FF, and metabolites. Detailed procedures are provided in Text S2 and S13. Additionally, to investigate the effects of pyridoxal on *A. pyrenoidosa*, a co-culture experiment among *Pseudomonas*_sp1, pyridoxal, and *A. pyrenoidosa* was also conducted, and relevant information was detailed in Text S14.

### Statistical analysis

The transcriptomic, metabolomic, metagenomic, and whole genome data were analyzed and visualized using the online Majorbio Cloud Platform [37]. One-way analysis of variance (ANOVA) or Kruskal–Wallis tests were used to evaluate the statistical difference among groups. The principal coordinate analysis (PCoA), principal component analysis (PCA), orthogonal partial least squares discrimination analysis (OPLS-DA), mental test, Procrustes test, and heatmap were performed in R 4.0.5 with different packages. The co-occurrence network was performed using Gephi 0.9.2 to visualize the potential hosts of ARGs [38]. SPSS 21.0 was used for all statistical analyses. GraphPad Prism Version 8.0 was used to plot the diagrams.

## Results

### Adaptability of algae-bacteria symbionts to PLA MPs and FF treatments

**Fig. 1:**
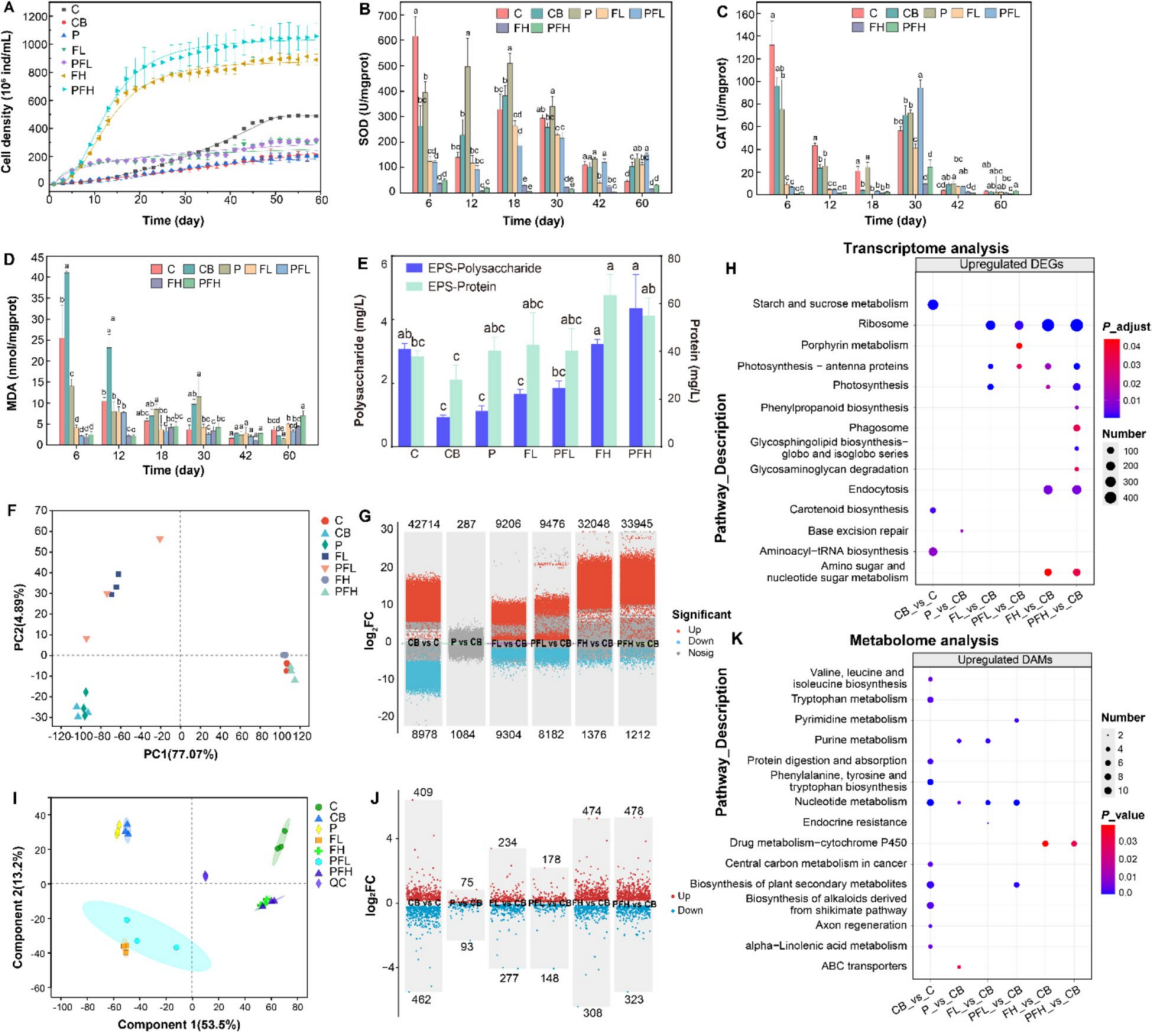
Response of A. pyrenoidosa at physiological, transcriptional, and metabolic levels under FF and PLA MPs treatments. **A** The growth curves of A. pyrenoidosa. **B**, **C**, **D** Changes in SOD activity (**B**), CAT activity (**C**), and MDA contents (**D**) of A. pyrenoidosa with incubation time. **E** EPS productions of A. pyrenoidosa in different groups. **F** PCA analysis of DEGs of A. pyrenoidosa in different groups. **G** Scatter plots displaying upregulated and downregulated DEGs. **H** KEGG enrichment analysis of upregulated DEGs. **I** OPLS-DA analysis of DAMs of A. pyrenoidosa in different groups. **J** Scatter plots displaying upregulated and downregulated DAMs. **K** KEGG enrichment analysis of upregulated DAMs. Different letters indicate significant differences between different groups (*P* < 0.05, one-way ANOVA)

The addition of exogenous bacteria significantly inhibited the growth of *A. pyrenoidosa* compared to axenic cultures (Fig. 1A). In algae-bacterial co-cultures, treatment with PLA MPs had no significant effect on the growth of *A. pyrenoidosa*, whereas single FF treatment and co-treatment with PLA MPs promoted algal growth in a dose- and time-dependent manner (Fig. 1A). Photosynthetic pigment content showed a similar variation pattern to algal density (Fig. S1). The contents of chlorophyll a (Chl-a), chlorophyll b (Chl-b), and carotenoids showed no significant difference compared to the control after PLA MPs treatment. However, these pigment contents exhibited a concentration-dependent increase after treatment with different concentrations of FF. At the end of the cultivation, the Chl-a content in the low-concentration and high-concentration FF treatments was approximately 2 times and 14 times that of the control group, respectively (Fig. S1). In addition, FF exposure resulted in significant reductions in superoxide dismutase (SOD) activity (Fig. 1B), catalase (CAT) activity (Fig. 1C), and malondialdehyde (MDA) content (Fig. 1D). Especially at the early stage of culture, the SOD, CAT, and MDA contents in the FF treatment groups decreased by 4–6 times, 14–47 times, and 14–17 times, respectively, compared to the control group. FF exposure also stimulated the secretion of EPS by 0.5–1.0 times (Fig. 1E). SEM observation further displayed that more algal cells were partially or completely covered by secretions in FF treatments compared with the control group (Fig. S2).

### Adaptation mechanism of A. pyrenoidosa to PLA MPs and FF treatments

Compared to the control group, PLA MPs treatment showed similar gene expression patterns and metabolic profiles, while low-dose and high-dose FF treatments showed distinctly different patterns (Fig. 1F, I). Scatter plots visualized the variation in DEGs and DAMs in each treatment (Fig. 1G, J), indicating that FF induced upregulation of numerous DEGs and an increase in the abundance of DAMs, especially for high-dose FF treatment. These changes reflected the molecular mechanisms underlying the adaptive regulation of microalgae to FF. For example, the GO functional enrichment analysis revealed that DEGs under FF treatment were significantly enriched in several key biological categories, including “binding”, “catalytic activity”, “membrane fraction”, “cellular fraction”, “metabolic process”, and “cellular process” (Fig. S3). KEGG enrichment analysis of DEGs further revealed that upregulated DEGs were mainly enriched in the ribosome, photosynthesis–antenna proteins, and photosynthesis pathways (Fig. 1H and Fig. S4A). Those genes encoding proteins involved in photosynthesis, including atp, pet, psb, psa, and LHC, were significantly upregulated after FF treatment in a dose-dependent manner (Fig. S5). These results implied that the regulation of photosynthesis in response to FF provided more energy and material basis for microalgal growth. FF-induced DAMs were correspondingly mainly enriched in pathways related to cell growth, such as “purine metabolism”, “pyrimidine metabolism”, and “nucleotide metabolism” (Fig. 1K and Fig. S4B).

Furthermore, the antioxidant system of microalgae is a survival mechanism to cope with stress. In our study, the expression of genes encoding antioxidant enzymes such as CAT, SOD, APX, GST, and GSR was upregulated to protect algal cells from oxidative stress damage after FF treatments (Fig. S6), which was consistent with the physiological results. The enrichment of some antioxidant compounds, such as ascorbic acid and glutathione, in *A. pyrenoidosa* after FF treatment also provided support for the antioxidant defense of microalgae (Fig. S7).

Notably, additional adaptive strategies were observed when *A. pyrenoidosa* were exposed to high-dose FF stress. The expression of genes involved in the endocytosis and ABC transporter pathways changed significantly in FH and PFH groups (Fig. 1H and Fig. S4A). Given that endocytosis can mediate the internalization of external substances (including potential bacterial signals), and ABC transporters are key for compound export and detoxification, these changes suggest a potential enhancement of microalgal–bacterial interactions under stress, possibly facilitating a cooperative response to FF exposure. The upregulated DAMs were enriched in the “drug metabolism-cytochrome P450” pathway (Fig. 1K), indicating that high-dose FF stimulated the metabolism of algal cells to exogenous pollutants.

### Changing in microalgae-associated microbes

**Fig. 2:**
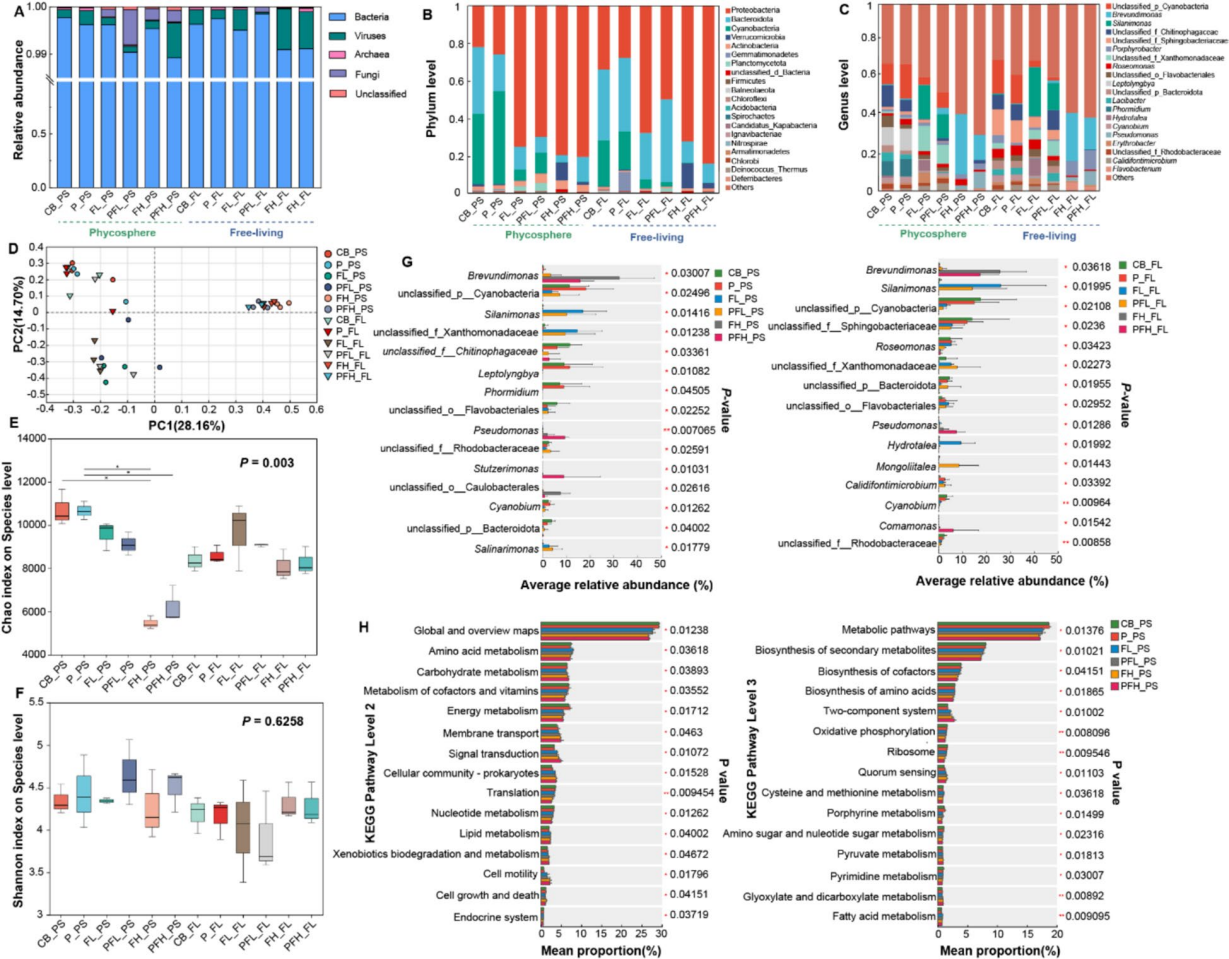
Composition and function of phycospheric and free-living bacteria. **A** The relative abundance of microbes. **B** The relative abundance of phycospheric and free-living bacteria at the phylum level. **C** The relative abundance of phycospheric and free-living bacteria at the genus level. **D** PCA analysis of phycospheric and free-living bacteria based on the species level. **E** The Chao index of bacteria at the species level in different treatment groups. **F** The Shannon index of bacteria on the species level in different treatment groups. **G** The relative abundance of the top 15 bacteria at the genus level in the PS and FL samples. **H** KEGG functional annotation of the bacterial genomes. *P < 0.05; **P < 0.01

In general, bacteria were most abundant in all samples compared to viruses and fungi (Fig. 2A). PLA MPs still did not show significant effects on the composition of PS and FL bacteria, while FF showed strong effects on both PS and FL bacteria (Fig. 2B, C). The PCA further confirmed the obvious distinction between groups with or without FF treatment (Fig. 2D). Especially in FH and PFH groups, there were significant decreases in PS bacterial species richness (Fig. 2E), while no significant change was observed in PS bacterial species diversity (Fig. 2F). For bacterial community composition, FF led to a marked decrease in Bacteroidetes and Cyanobacteria, with Proteobacteria becoming the dominant phylum (Fig. 2B). At the genus level, FF induced reductions in unclassified_p_Cyanobacteria, *Leptolyngbya*, *Phormidium*, and unclassified_f_Vhitinophagaceae (Fig. 2C). While unclassified_f_Xanthomonadaceae and *Silanimonas* were increased in FL and PFL groups, and *Brevundimonas* and *Pseudomonas* were increased in FH and PFH groups (Fig. 2G). Moreover, we found a decrease in the relative abundance of functional genes associated with normal physiological metabolism, including cofactor and vitamin metabolism, energy metabolism, and nucleotide metabolism, and an increase in the relative abundance of functional genes involved in membrane transport, signal transduction, cell motility, two-component system, and quorum sensing under FF treatment (Fig. 2H). These results suggested that FF-induced enrichment of specific bacteria reduced their survival costs and enhanced the communication with microalgal cells, thus better cooperating with microalgae to cope with environmental stress.

### Proliferation of ARGs in the phycosphere

**Fig. 3:**
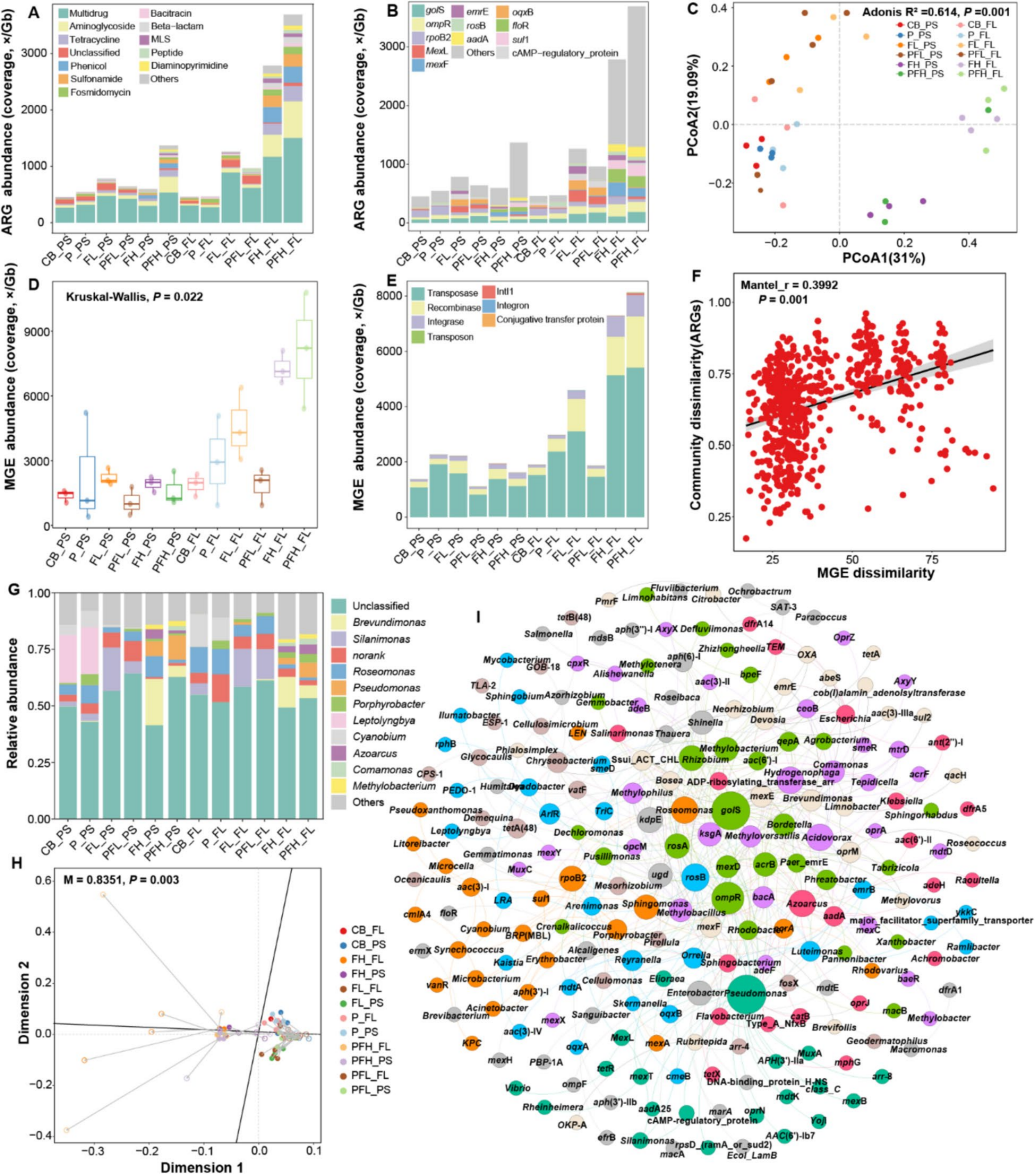
Antibiotic resistome in the phycosphere and free-living environment under PLA MPs and FF treatments. **A** The abundance of the top 12 ARG types in each group. **B** The abundance of the top 12 ARG subtypes in each group. **C** PCoA analysis of ARGs within the phycosphere and free-living environment. **D** The abundance of MGEs co-occurring with ARGs in each group. **E** The abundance of MGE types co-occurring with ARGs in each group. **F** Linear regression analysis between ARGs and MGEs. **G** The relative abundance distribution of the top 12 ARG hosts at the genus level in each group. **H** Procrustes analysis between ARG subtypes and hosts at the genus level. **I** Co-occurrence network analysis of ARG subtypes with hosts

A total of 18 ARG types were classified in all samples, comprising 186 ARG subtypes. Multidrug was the most dominant type in all groups, followed by aminoglycoside and tetracycline (Fig. 3A). PLA MPs treatment did not significantly affect the distribution of ARGs in FL and PS samples. Whereas FF and co-treatment increased the abundance of ARGs, which was positively correlated with FF concentration. In addition to the proliferation of phenicol-related ARGs, such as *flo*R, some non-corresponding ARGs, such as *gol*S, *Mex*L, *mex*F, and *oqx*B, were also enriched (Fig. 3B). The PCoA result also revealed significant changes in ARG profiles under FF treatment and a clear separation of ARG profiles between phycosphere and free-living environment (*R*^2^ = 0.614, *P* = 0.001, Fig. 3C).

To explore the potential mobility of ARGs under different treatments, the co-occurrence analysis was performed for contigs carrying both ARGs and MGEs. The results showed that FF did not induce significant changes in MGEs in the phycosphere but induced a significant increase in the free-living environment (Kruskal–Wallis, *P* = 0.022) (Fig. 3D). Transposase was the most dominant MGE type in all groups, followed by recombinase and integrase (Fig. 3E). FF mainly induced increases in these three MGE types in FL samples. As MGEs-mediated horizontal gene transfer played a significant role in the transmission of ARGs, the significant correlation between MGEs and ARGs further demonstrated the important contribution of MGEs to the proliferation of ARGs in the free-living environment (Mantel_r = 0.3992, *P* = 0.001) (Fig. 3F).

In addition, changes in ARGs’ hosts can directly reflect changes in ARGs’ profiles under FF and PLA MPs’ treatment. Consistent with shifts in bacterial communities, FF treatment induced an increase in the abundance of Proteobacteria hosts at the phylum level, particularly from approximately 40% in the control to over 75% under low-dose FF (Fig. S8). At the genus level, low-dose FF increased the abundance of *Silanimonas* hosts by approximately 2 to 10 times, and high-dose FF induced *Brevundimonas* and *Pseudomonas* to become the dominant hosts (Fig. 3G). Procrustes analysis indicated a significant correlation between ARG subtypes and hosts (Procrustes *M*^2^ = 0.8351, *P* = 0.003) (Fig. 3H). Therefore, network analysis was further used to visually reveal their co-occurrence relationship. As shown in Fig. 3I, those dominant hosts, including *Silanimonas*, *Pseudomonas*, and *Brevundimonas*, were found to potentially carry multiple ARGs, including *Mex*L, *mex*F, *tet*R, and *aad*A25. Meanwhile, some ARG subtypes, such as *gol*S, *omp*R, *rpo*B2, and *mex*F, were also found to have multiple potential hosts. These results suggested that the enrichment of these hosts in the phycosphere after FF and PLA MPs’ treatment contributed to the increase and spread of ARGs.

### The relationship between microalgae, bacteria, and ARGs in the phycosphere

**Fig. 4:**
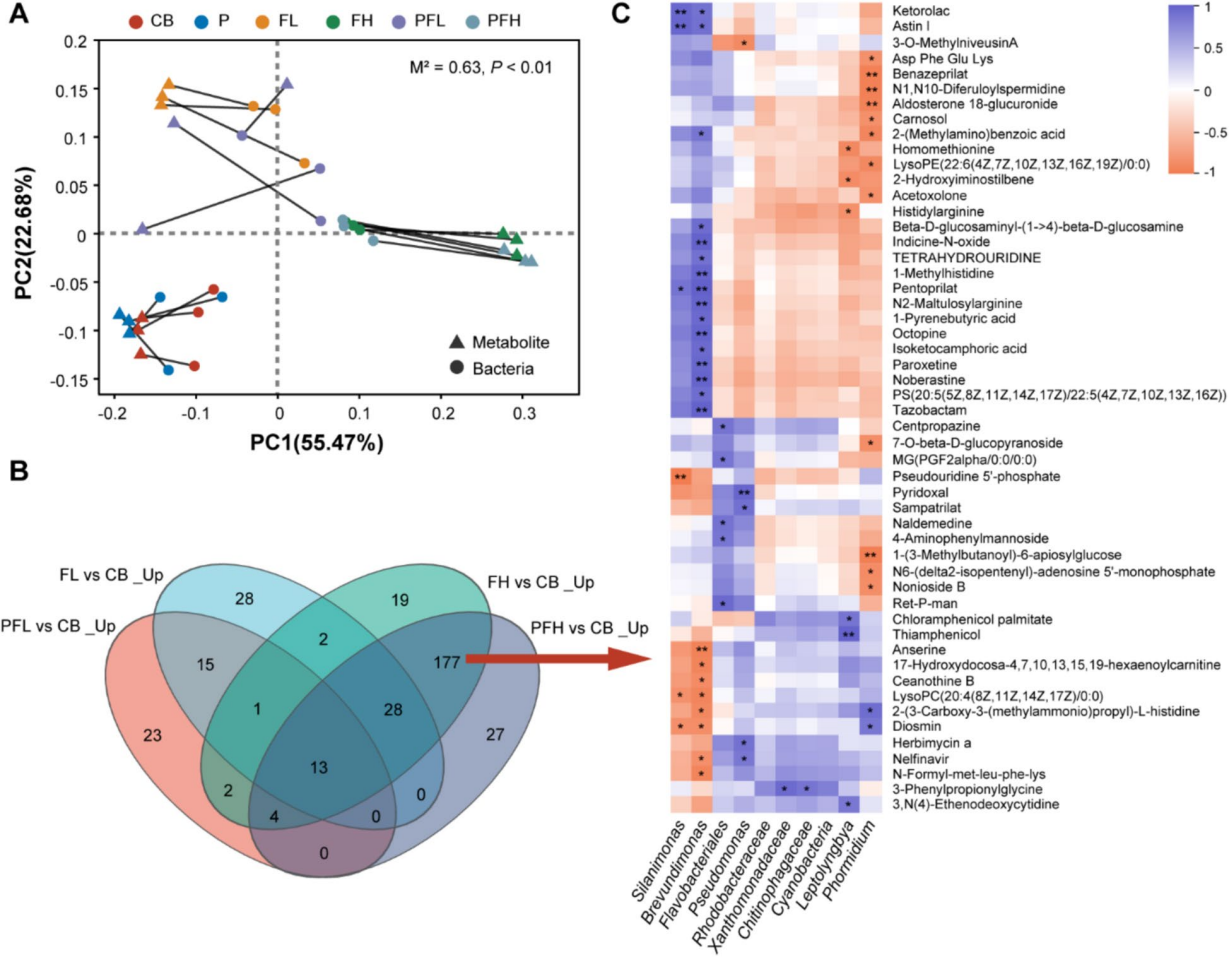
The association analysis of metabolome and bacterial community under PLA MPs and FF treatment. **A** Procrustes analysis between DAMs and bacteria at the genus level. **B** Venn diagram of upregulated DAMs (_Up) in the four FF treatments. **C** The correlation heatmap between selected DAMs and phycospheric bacteria. *P < 0.05; **P < 0.01

Procrustes test combined with PCoA analysis showed a significant correlation between metabolic profiles and bacterial communities (*P* < 0.05) (Fig. 4A), indicating that phycospheric metabolites might be important factors driving changes in the composition of the phycospheric bacterial community. Besides, our aforementioned results revealed the important contribution of bacterial communities to ARGs in the phycosphere under FF and PLA MPs’ treatment and the significant proliferation of ARGs in the PFH group. Therefore, the DAMs in the PFH group were selected for further analysis of the relationship between metabolites and phycospheric bacteria (Fig. 4B). It was clear from the correlation heatmap that *Brevundimonas*, *Flavobacteriales*, *Pseudomonas*, and *Phormidium* were significantly associated with many upregulated metabolites in the PFH group (Fig. 4C). This implied that the enrichment of these bacteria, including dominant ARG hosts in the phycosphere, was most likely regulated by the increase in the associated metabolites. Together, in addition to the direct selective effect of environmental stress on bacterial communities, stress-induced changes in microalgal metabolism could affect the enrichment of bacterial communities in the phycosphere, which indirectly affects the distribution of phycospheric ARGs.

### Whole genome sequencing of dominant ARG hosts

**Fig. 5:**
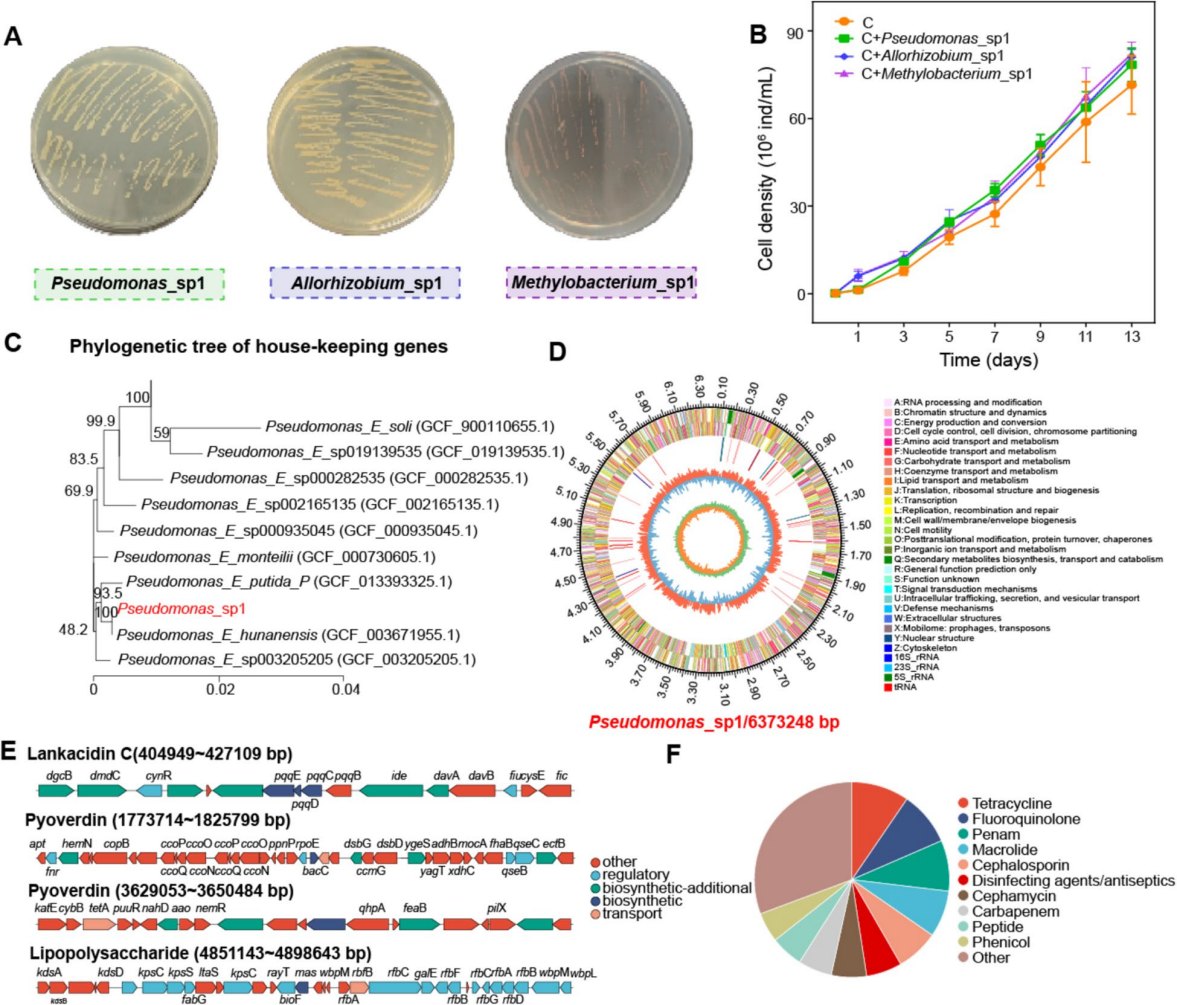
Isolation of dominant ARG hosts and genomic information of Pseudomonas_sp1. **A** Isolation of dominant ARG hosts using the plate coating method. **B** Growth curve of A. pyrenoidosa co-cultured with Pseudomonas_sp1, Allorhizobium_sp1, Methylobacteium_sp1. **C** Phylogenetic tree of Pseudomonas_sp1 based on housekeeping genes. Numbers listed at the branches were bootstrap values. **D** Genetic map of Pseudomonas_sp1. From the outside to the inner: Circle 1, the genome size. Circles 2 and 3, protein-coding regions by COG function categories on forward/reverse strand, different categories of COG pathways were shown using different colors. Circle 4, the distribution of rRNA and tRNA. Circle 5, the GC content, the outer red part indicates that the GC content of this region is higher than the average GC content of the whole genome, and the inner blue part is the opposite. Circle 6, the GC-Skew value (G-C/G+C). **E** Linear map of predicted secondary metabolite synthesis gene clusters. **F** Prediction of antibiotic resistance gene types

Potential ARG hosts were isolated and screened from the phycosphere. After co-culturing with these isolates, whole genome sequencing and annotation of biosynthetic gene clusters (BGCs) were conducted to reveal their environmental adaptations. A total of three potential ARG hosts were screened and identified as *Pseudomonas*_sp1, *Allorhizobium*_sp1, and *Methylobacterium*_sp1 (Fig. 5A, C and Fig. S9A). All three isolates could promote the growth of *A. pyrenoidosa* (Fig. 5B). Most genes in their genomes were involved in membrane transport, signal transduction, amino acid metabolism, carbohydrate metabolism, as well as the metabolism of coenzyme factors and vitamins (Fig. 5D, Fig. S9B, and Fig. S10–S12). To provide more information on microalgae-bacteria interactions, we also annotated the genome for pathways involved in the biosynthesis of the metabolite vitamin B6, which was found to be significantly associated with the dominant ARG host *Pseudomonas* in the aforementioned results (Fig. 3C). The results showed that *Pseudomonas*_sp1, but not the other two strains, could further synthesize vitamin B6 via the pentose phosphate pathway (Fig. S13). Analysis of BGCs revealed the potential of these strains to produce compounds with quorum sensing, such as hserlactone (Fig. 5E, Fig. S9C, and Table S5–S7). Specifically, *Pseudomonas*_sp1 also had the potential to produce compounds with antimicrobial activity, such as pyoverdine and lankacidin C (Fig. 5E). The prediction of ARGs indicated that these bacteria contained multiple ARGs, and *Pseudomonas*_sp1 contained more resistance gene types than the other two strains (Fig. 5F and S9D). These findings provided strong support for the dominant position of *Pseudomonas* in the phycosphere.

### Role of specific-enriched Pseudomonas sp. in the phycosphere

**Fig. 6:**
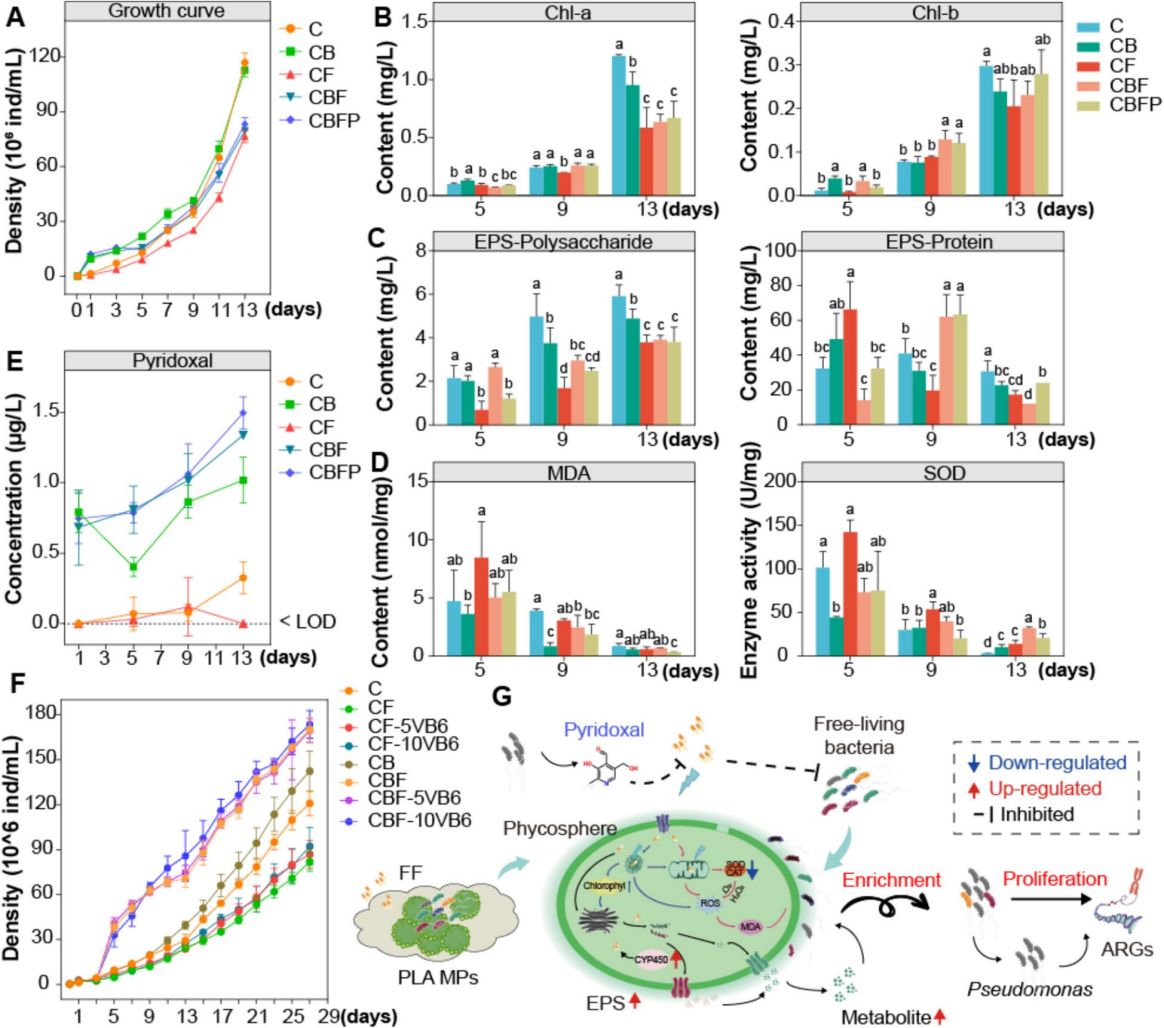
Physiological responses of A. pyrenoidosa in coculture with Pseudomonas_sp1 under single FF or combination with PLA MPs treatment. **A** The growth curves of A. pyrenoidosa co-cultured with Pseudomonas_sp1 in different groups. **B** The content of chlorophyll of A. pyrenoidosa in different groups. **C** The EPS contents of A. pyrenoidosa in different groups. **D** The MDA content and SOD activity of A. pyrenoidosa in different groups. **E** The content of pyridoxal in the culture medium of different groups. **F** Growth curves of A. pyrenoidosa co-cultured with Pseudomonas_sp1 and/or pyridoxal in different groups. **G** Conceptual diagram of the mechanism by which Pseudomonas sp. enhances the adaptability of A. pyrenoidosa under FF and PLA MPs stress by secreting vitamin pyridoxal, thereby establishing its competitive advantage within the phycosphere and ultimately promoting the proliferation and dissemination of ARGs. Different lowercase letters (a, b, c, d) above the bars indicate significant differences between different groups (*P* < 0.05, one-way ANOVA)

Our aforementioned results indicated that *Pseudomonas* significantly increased and became the dominant ARG host after high-dose FF exposure (Figs. 2 and 3). The isolated *Pseudomonas*_sp1 also showed strong resistance to FF in the MIC test (Table S8). Analysis of its biological characteristics also revealed that *Pseudomonas*_sp1 had stronger colonization advantages in the phycosphere. Therefore, *Pseudomonas*_sp1 was selected for co-cultivation with axenic *A. pyrenoidosa* to explore how stress-selected bacteria cooperate with microalgae in the phycosphere. The results showed that compared to axenic microalgae, co-cultures with *Pseudomonas*_sp1 promoted microalgal growth within 13 days of incubation (Fig. 6A). Axenic microalgae showed remarkable growth inhibition under FF treatment. After inoculating *Pseudomonas*_sp1, FF stress on microalgae was alleviated (Fig. 6A). These results confirmed the positive effect of *Pseudomonas*_sp1 on stress adaptation in *A. pyrenoidosa*.

Furthermore, we focused on how microalgae improved their adaptability to FF stress when co-cultured with phycospheric bacteria. Firstly, no significant changes in FF concentration in all treatments were observed after 13 days of incubation (Table S9). Under FF treatment, microalgae co-cultured with *Pseudomonas*_sp1 exhibited significant physiological changes compared to the axenic microalgae, with chlorophyll content increased by approximately 0.35 times (Fig. 6B) and EPS production increased by about 1.9 times (Fig. 6C). The decrease in MDA content and SOD activity indicated that the presence of *Pseudomonas*_sp1 reduced oxidative stress caused by FF (Fig. 6D). These responses were the adaptive manifestations of microalgae after inoculation with *Pseudomonas*_sp1. In addition, our aforementioned results indicated a significant positive correlation between FF-mediated changes in the abundance of *Pseudomonas*_sp1 and metabolites such as pyridoxal, one of the forms of vitamin B6. Therefore, in the co-culture experiment, the production of pyridoxal was also recorded in each group. It was found that pyridoxal was detected only in the presence of *Pseudomonas*_sp1 and gradually increased with the extension of culture time (Fig. 6E), indicating that *Pseudomonas*_sp1 was capable of producing pyridoxal. FF stress further stimulated the production of pyridoxal compared to the non-FF treatment. Another co-culture experiment further showed that pyridoxal (5 and 10 μg/L) can promote the growth of axenic *A. pyrenoidosa* with dose effects, especially in the presence of *Pseudomonas*_sp1 (Fig. 6F).

## Discussion

In aquatic environments, phytoplankton are facing stress from a variety of pollutants, such as antibiotics and microplastics, due to high-intensity human activities [3, 39]. Recent studies showed that microalgae could also exhibit strong adaptability and resistance to abiotic stresses like terrestrial plants [6, 40, 41], but the basic mechanism underlying this phenomenon remains unclear. In this study, we described that FF and PLA MPs not only stimulated the self-defense system of *A. pyrenoidosa* but also affected the growth of microalgae and the occurrence of ARGs by reshaping microalgae-associated microbes. These findings provided in-depth insights into the contribution of microalgae-associated microbes to the adaptation of microalgae to abiotic stresses and the distribution of ARGs.

Previous studies showed that in order to adapt to external stresses, plants have developed a series of self-defense mechanisms, mainly including regulation, defense, and metabolic accumulation [42, 43]. In our study, similar response mechanisms were observed in microalgae under single FF treatment and co-treatment with PLA MPs. The expression levels of genes involved in the regulation of photosynthesis and nucleotide metabolism were significantly increased (Fig. 1H and Fig. S5), indicating that FF and PLA MPs stimulated photosynthesis in *A. pyrenoidosa* and promoted their growth. Meanwhile, the antioxidant response revealed a dynamic adaptation process. The initial decrease in antioxidant enzyme activities might indicate that the ROS burst was induced under FF exposure (Fig. 1B, C). The subsequent upregulation of related enzyme genes’ expression in *A. pyrenoidosa* (Fig. S6) suggested the activation of a long-term antioxidant defense system to restore the redox balance. It was worth noting that although excessive ROS was known to cause potential oxidative damage, low levels of ROS might exhibit a hormesis effect on microalgal growth [44]. Under high-dose FF exposure, *A. pyrenoidosa* also stimulated the activity of cytochrome P450 enzymes (Fig. 1K), which played critical roles in both the metabolism of endogenous compounds and detoxification of xenobiotics [45], thereby alleviating the stress of FF. In terms of metabolite accumulation, the enrichment of EPS and antioxidant metabolites such as glutathione and ascorbic acid played important roles in improving microalgal resistance to FF and PLA MPs’ stress [44, 46]. The secreted EPS could form a protective layer on the cell surface, which can effectively adsorb and immobilize pollutants, thereby reducing their direct contact with and penetration into the cells. This physical barrier function not only alleviated immediate toxicity but also provided a stable microenvironment for the detoxification and antioxidant systems [47]. As a result, *A. pyrenoidosa* showed strong self-adaptation to pollutant stress.

Our previous work found that exogenous pollutants did not affect the distribution of core microbiota in the phycosphere when microalgae were in the exponential growth phase [17]. However, in this study, recruitment of different bacterial species appeared in the phycosphere after microalgal growth entered a stable phase under FF and PLA MPs treatments. The distinct recruitment of *Pseudomonas* sp. in this study, compared to our previous findings [17], can likely be attributed to the extended duration of the experiment and the subsequent entry of *A. pyrenoidosa* into the stationary phase. During this phase, altered algal exudates, as evidenced by our metabolomic data, may create a niche that selectively enriches for bacteria such as *Pseudomonas*, which are adept at utilizing these specific substrates and providing essential metabolites like vitamin B6 in return [48, 49]. It’s been proven by previous studies that changes in the metabolic activities of microalgae in different periods may provide suitable living conditions and nutrients for newly colonized bacteria [50, 51]. Furthermore, despite the reduced alpha diversity of the recruited bacteria, a consistent increase in those bacteria mediated by FF and PLA MPs was observed (Fig. 1). This could be explained by the fact that when challenged with FF and PLA MPs, microalgae mostly recruited growth-promoting beneficial bacteria and inhibited the recruitment of harmful bacteria [6, 52]. This change is a form of niche filling based on mutual adaptation between microalgae and bacteria and optimization for improved nutrient utilization [53]. For example, the significant reduction in unclassified_p_Cyanobacteria might be attributed to competition for nutrients and growth space between them and *A. pyrenoidosa* under external stress [23]. The obvious increase of *Pseudomonas* might be attributed to their potential resistance to FF [54], and they have been commonly found to be beneficial symbiotic bacteria of *Chlorella* sp. in many studies, particularly adept at acquiring phytoplankton-derived dissolved organic matter [55, 56]. In our study, *Pseudomonas* isolates were also found to be multi-antibiotic resistant and have the unique biosynthetic potential to produce pyoverdine and potentially antimicrobial molecules, such as lankacidin C (Fig. 5E), which was beneficial to maintain their colonization and facilitate microalgal growth [57–59]. Therefore, in addition to the adaptive strategies developed by microalgae themselves to cope with pollutant stress, stress-induced recruitment of specific bacteria is also an effective mechanism that cannot be ignored [18, 52].

Notably, different from the stress caused by environmental factors such as temperature and salinity, antibiotic stress is also the direct cause of ARGs [60]. In our study, single FF treatment and co-treatment with PLA MPs, especially at high-dose FF, induced the proliferation of both corresponding and non-corresponding ARGs (Fig. 3A). Many studies revealed that cross-selection of antibiotics was prevalent, and many exogenous pressures weren’t on certain unique or specific ARGs, but on the resistome due to the concurrence of ARGs in MGEs [30, 61]. In addition, the strength of the co-selection effect is often concentration-dependent [11], which is consistent with our results that the abundance of ARGs under high-dose FF exposure was much higher than that under low-dose FF exposure. Vertical transmission of ARGs and MGEs-mediated horizontal gene transfer are two important ways of ARGs proliferation [30, 62]. FF treatment induced a significant increase in MGEs co-occurring with ARGs in the free-living environment but not in the phycosphere (Fig. 3D). This might stem from the selection pressure of different habitats and the differences in ecological processes. Free-living bacteria directly exposed to FF might rely more on rapidly acquiring resistance through horizontal gene transfer. However, in the phycosphere, it is possible to adapt to stress by screening and enriching specific ARG hosts. Further tracing of ARG hosts revealed the main contribution of bacterial communities to the development of phycospheric ARGs. FF induced more diverse ARGs hosts, such as *Silanimonas*, *Brevundimonas*, and *Pseudomonas*, which were the dominant bacteria colonized in the phycosphere under FF treatment (Fig. 3G). Thus, FF-mediated selectivity in the phycospheric bacteria simultaneously caused the proliferation of phycospheric ARGs. Because of developing multiple ARG types under antibiotic stress, these bacteria might be potentially resistant to multiple antibiotics, and they were found to be important participants in ARGs transmission in previous studies [63, 64], which all implied an increased ecological risk of ARGs in aquatic environments.

As aforementioned, the reconstruction of phycospheric microbes under antibiotic stress played a key role in improving the adaptability of microalgae and enrichment of phycospheric ARGs. However, it was striking that our metagenomic results also showed that despite the consistent growth-promoting effect of FF-induced phycospheric bacteria, there were differences in the core-associated microbes recruited by microalgae under different concentrations of FF treatment. For instance, we found that unclassified_f_Xanthomonadaceae and *Silanimonas* were enriched in FL and PFL, while *Brevundimonas* and *Pseudomonas* were enriched in FH and PFH (Fig. 2G). Besides, the phycosphere showed a different pattern of ARGs enrichment compared to the free-living environment. These results implied that microalgae modulated microalgae-bacteria interactions through different bacterial recruitment strategies to help them adapt to different levels of stress. Bacteria are critically dependent on the dissolved organic matter secreted by microalgae to support their growth and use motility, chemotaxis, or attachment to colonize the phycosphere [18]. Meanwhile, microalgae have an innate ability to actively regulate their colonizing bacteria, releasing certain unique metabolites to facilitate association with potentially beneficial bacteria [22]. Our results showed significant differences in the metabolic profiles of microalgae when exposed to different concentrations of FF (Fig. 1I), as well as significant correlations between metabolites and phycospheric bacteria (Fig. 4A), indicating that different concentrations of FF probably mediated the alteration of microalgal exudate to construct specific microalgae-associated microbes, in turn driving positive feedbacks on microalgal growth and defense. However, it is still not well understood which specific compounds among the complex metabolites drive the colonization of beneficial bacteria.

The environmental adaptability of bacteria and their interaction with microalgae jointly determine their colonization in the phycosphere [65, 66]. Our study demonstrated that the core bacteria, *Pseudomonas*, had great advantages in improving the adaptability and resistance of microalgae to FF stress; further attention to it would help reveal how stress-selected phycospheric bacteria cooperate with microalgae. The results showed that *Pseudomonas* isolates promoted photosynthetic pigment synthesis, increased antioxidant activity, and stimulated EPS production in *A. pyrenoidosa* (Fig. 6A–D), all of which were important ways for *A. pyrenoidosa* to adapt to unfavorable conditions [44, 46]. Co-culture of *Pseudomonas* isolates with *A. pyrenoidosa* did not enhance microalgal adaptation by degrading antibiotics, as reported in previous studies [4, 40], and FF-stimulated production of vitamin B6 most likely played an important role. Vitamin B6, as a cofactor for many enzymatic reactions, is a vital player in protein and amino acid metabolism [67]. Its increase could promote the metabolic reaction of algal cells to achieve the promotion of algal growth. Our experimental data from the pyridoxal supplementation co-culture experiment (Fig. 6F) provide direct evidence supporting this mechanism, demonstrating that exogenous vitamin B6 alone is sufficient to promote axenic algal growth, with a synergistic effect observed in the presence of *Pseudomonas*_sp1. This finding significantly strengthens the causal link between *Pseudomonas*-derived vitamin B6 and the enhanced fitness of *A. pyrenoidosa* under stress. Meanwhile, vitamin B6 as a nutrient might provide a more favorable phycosphere environment for the growth of other bacteria [68]. Previous studies also reported that *Pseudomonas* could produce the plant hormone indole-3-acetic acid to promote algal growth [69, 70]. In algae-bacterial symbionts, compounds such as auxin [71], plant hormones [72], quorum-sensing signaling molecules [73], and bioactive secondary metabolites [22] are chemical signals of interspecific or intraspecific communication, and their role in regulating microalgae and associated bacterial communities cannot be ignored [40, 74].

In conclusion, our study advances our understanding of microalgal–bacterial interactions under pollutant stress in several key aspects: (1) it reveals that long-term exposure during the stationary phase leads to a distinct phycosphere microbiome assembly compared to short-term exponential phase studies. (2) Through multi-omics sequencing, we demonstrate that pollutant-induced changes in algal exudates are a primary driver for enriching specific antibiotic-resistant bacteria (e.g., *Pseudomonas*), which in turn facilitates ARGs’ propagation within the phycosphere. (3) We move beyond correlation by isolating the dominant bacterial host and experimentally confirming its role in enhancing algal fitness, providing strong mechanistic evidence for a mutualistic relationship mediated potentially by vitamin B6 (Fig. 6G), an insight not previously reported in this context. However, it should be acknowledged that the use of unaged PLA MPs in this study may not fully capture the ecological effects of environmentally relevant, aged microplastics. This might partially explain why the observed direct effects of these microplastics in our study were negligible. Additionally, although controlled laboratory conditions are crucial for mechanism research, they may not encompass all the complexities of the natural aquatic environment. Future studies that employ aged microplastics and more complex environmental simulations will be valuable to further validate and contextualize these findings.

## Conclusion

Our study demonstrates that exposure to florfenicol and polylactic acid microplastics induces significant adaptive responses in *A. pyrenoidosa*, enhancing its growth and antioxidant capacity through transcriptional and metabolic reprogramming. Concurrently, these stressors reshape the phycosphere microbiome, enriching antibiotic-resistant bacteria, particularly *Pseudomonas* sp., which establishes its competitive advantage by secreting vitamin B6 (pyridoxal) to facilitate algal adaptation. This mutualistic interaction underscores the role of cross-kingdom chemical mediation in mitigating abiotic stress. Furthermore, the proliferation of ARGs is driven primarily by the propagation of resistant host bacteria rather than horizontal gene transfer, highlighting an ecological risk in polluted aquatic systems. Our study provides support for understanding that the mutualistic symbiosis between microalgae and bacteria, driven by chemical mediators, enhances the adaptability and resistance of microalgae to abiotic stress.

## Supplementary Information

Supplementary Material 1: Text S1. Details of the pre-experiment. Text S2. Determination of physiological properties of A. pyrenoidosa. Text S3. Transcriptomic analysis. Text S4. Non-target metabolomic analysis. Text S5. Metagenomic analysis. Text S6. Calculation of ARGs’ARGs abundance. Text S7. Identification of ARC hosts. Text S8. Identification of MGEs. Text S9. Isolation of phycospheric bacteria. Text S10. Whole genome sequencing of phycospheric isolates. Text S11. Determination of the minimum inhibitory concentration (MIC) of phycospheric isolates on FF. Text S12. The co-culture experiment of phycospheric isolates and A. pyrenoidosa. Text S13. Quantification of FF and the metabolite pyridoxal. Text S14. Co-culture of Pseudomonas_sp1, pyridoxal, and A. pyrenoidosa. Figure S1 Changes in photosynthetic pigment content of A. pyrenoidosa under PLA MPs and FF treatment. Figure S2 Morphological characteristics of PLA MPs and A. pyrenoidosa. Figure S3 GO functional classification of upregulated (A) and downregulated DEGs (B). Figure S4 KEGG enrichment analysis of downregulated DEGs (A) and DAMs (B). Figure S5 Heatmaps of DEGs related to photosynthesis (A) and antenna proteins (B). Figure S6 Heatmap of DEGs related to antioxidation. Figure S7 Relative abundance of DAMs associated with antioxidation in different treatment groups. Figure S8 The relative abundance distribution of the top 12 ARG hosts at the phylum level in each group. Figure S9 Genomic information of Allorhizobium_sp1 and Methylobacteium_sp1. Figure S10 Annotation of KEGG pathways for strain Pseudomonas_sp1 genome. Figure S11 Annotation of KEGG pathways for strain Allorhizobium_sp1 genome. Figure S12 Annotation of KEGG pathways for strain Methylobacteium_sp1 genome. Figure S13 Pathways of vitamin B6 metabolism. Figure S14 UPLC–MS/MSUPLC-MS/MS chromatograms of FF, FF-D3, pyridoxal, and pyridoxal-D3. Table S1. The concentration of FF in the medium of each group in the pre-experiment. Table S2. Mobile phase elution gradients. Table S3. Grouping for co-culture experiment of phycospheric isolates and A. pyrenoidosa. Table S4. UPLC–MS/MSUPLC-MS/MS conditions for FF, FF-D3, pyridoxal, and pyridoxal-D3 determination. Table S5. The table of secondary metabolites synthesis gene clusters in Pseudomonas_sp1. Table S6. The table of secondary metabolites’metabolites synthesis gene clusters in Methylobacterium_sp1. Table S7. The table of secondary metabolites’metabolites synthesis gene clusters in Allorhizobium_sp1. Table S8. The minimum inhibitory concentration (MIC) of three phycospheric bacteria isolates. Table S9. The concentration of FF in the medium of each group in the co-culture experiment of Pseudomonas_sp1 and axenic A. pyrenoidosa. Table S10 Groups for pyridoxal addition experiments.

## Data Availability

All data generated or analyzed during this study are included in this published article and its additional files and can be accessed via the databases listed below. The raw data of the transcriptome sequence have been deposited in the National Center of Biotechnology Information (NCBI) Sequence Read Archive (SRA) database (BioProject: PRJNA1171622, [https://www.ncbi.nlm.nih.gov/bioproject/PRJNA1171622] (https:/www.ncbi.nlm.nih.gov/bioproject/PRJNA1171622)); the raw data of the metagenome sequence have been deposited in the SRA database (BioProject: PRJNA1027504, [https://www.ncbi.nlm.nih.gov/bioproject/PRJNA1027504] (https:/www.ncbi.nlm.nih.gov/bioproject/PRJNA1027504)); the raw data of the whole genome sequence have been deposited in the SRA database for *Pseudomonas* _sp1 (BioProject: PRJNA1171391, [https://www.ncbi.nlm.nih.gov/bioproject/PRJNA1171391] (https:/www.ncbi.nlm.nih.gov/bioproject/PRJNA1171391)), *Methylobacterium* _sp1 (BioProject: PRJNA1171480, [https://www.ncbi.nlm.nih.gov/bioproject/PRJNA1171480] (https:/www.ncbi.nlm.nih.gov/bioproject/PRJNA1171480)), and *Allorhizobium* _sp1 (BioProject: PRJNA1171486, [https://www.ncbi.nlm.nih.gov/bioproject/PRJNA1171486] (https:/www.ncbi.nlm.nih.gov/bioproject/PRJNA1171486)); the raw data of the metabolome sequence have been deposited in the MetaboLights database under the accession number MTBLS11371 (https://www.ebi.ac.uk/metabolights/editor/MTBLS11371).
