# Supplementary material for: Stress-induced enrichment of Pseudomonas sp. stimulates the adaptive response of Auxenochlorella pyrenoidosa and antibiotic-resistant proliferation
Source: Microbiome. 2026 Feb 23;14:99. doi: 10.1186/s40168-026-02335-7 (PMC13037320; doi:10.1186/s40168-026-02335-7)
Supplement: Supplementary file 2 — Supplementary Material 1: Text S1. Details of the pre-experiment. Text S2. Determination of physiological properties of A. pyrenoidosa. Text S3. Transcriptomic analysis. Text S4. Non-target metabolomic analysis. Text S5. Metagenomic analysis. Text S6. Calculation of ARGs’ARGs abundance. Text S7. Identification of ARC hosts. Text S8. Identification of MGEs. Text S9. Isolation of phycospheric bacteria. Text S10. Whole genome sequencing of phycospheric isolates. Text S11. Determination of the minimum inhibitory concentration (MIC) of phycospheric isolates on FF. Text S12. The co-culture experiment of phycospheric isolates and A. pyrenoidosa. Text S13. Quantification of FF and the metabolite pyridoxal. Text S14. Co-culture of Pseudomonas_sp1, pyridoxal, and A. pyrenoidosa. Figure S1 Changes in photosynthetic pigment content of A. pyrenoidosa under PLA MPs and FF treatment. Figure S2 Morphological characteristics of PLA MPs and A. pyrenoidosa. Figure S3 GO functional classification of upregulated (A) and downregulated DEGs (B). Figure S4 KEGG enrichment analysis of downregulated DEGs (A) and DAMs (B). Figure S5 Heatmaps of DEGs related to photosynthesis (A) and antenna proteins (B). Figure S6 Heatmap of DEGs related to antioxidation. Figure S7 Relative abundance of DAMs associated with antioxidation in different treatment groups. Figure S8 The relative abundance distribution of the top 12 ARG hosts at the phylum level in each group. Figure S9 Genomic information of Allorhizobium_sp1 and Methylobacteium_sp1. Figure S10 Annotation of KEGG pathways for strain Pseudomonas_sp1 genome. Figure S11 Annotation of KEGG pathways for strain Allorhizobium_sp1 genome. Figure S12 Annotation of KEGG pathways for strain Methylobacteium_sp1 genome. Figure S13 Pathways of vitamin B6 metabolism. Figure S14 UPLC–MS/MSUPLC-MS/MS chromatograms of FF, FF-D3, pyridoxal, and pyridoxal-D3. Table S1. The concentration of FF in the medium of each group in the pre-experiment. Table S2. Mobile phase e [file 40168_2026_2335_MOESM1_ESM.docx]

**Supporting Information for**

**Stress-induced enrichment of *Pseudomonas* sp. stimulates the adaptive response of *Auxenochlorella pyrenoidosa* and antibiotic-resistant proliferation**

Qian Liu^1,2^, Jia Jia^1,*^, Xin Chen^1,3^, Chenxi Wu^1,4^

1. Aquatic Biodiversity and Water Ecological Environment Protection Research Center, Institute of Hydrobiology, Chinese Academy of Sciences, Wuhan, 430072, China

2. Nanchang Normal University, Nanchang 330101, China

3. Institute of Chemistry, Hubei Institute of Measurement and Testing Technology, Wuhan, 430073, China

4. Qinghai Lake Comprehensive Observation and Research Station, Chinese Academy of Sciences, Haibei 812200, China

*Corresponding author

Jia Jia

Email: [jia263319@ihb.ac.cn](mailto:jia263319@ihb.ac.cn)

Address: Donghu South Road #7, Wuhan, 430072, P. R. China

**Number of Texts: 14**

**Number of Figures: 14**

**Number of Tables: 10**

**Text captions**

**Text S1.** Details of the pre-experiment

**Text S2.** Determination of physiological properties of *A. pyrenoidosa*

**Text S3.** Transcriptomic analysis

**Text S4.** Non-target metabolomic analysis

**Text S5.** Metagenomic analysis

**Text S6.** Calculation of ARGs abundance

**Text S7.** Identification of ARC hosts

**Text S8.** Identification of MGEs

**Text S9.** Isolation of phycospheric bacteria

**Text S10.** Whole genome sequencing of phycospheric isolates

**Text S11.** Determination of the minimum inhibitory concentration (MIC) of phycospheric isolates on FF

**Text S12.** The co-culture experiment of phycospheric isolates and *A. pyrenoidosa*

**Text S13.** Quantification of FF and the metabolite pyridoxal

**Text S14.** Co-culture of *Pseudomonas*_sp1, pyridoxal and *A. pyrenoidosa*

**Figure captions**

**Fig. S1** Changes in photosynthetic pigment content of *A. pyrenoidosa* under PLA MPs and FF treatment.

**Fig. S2** Morphological characteristics of PLA MPs and *A. pyrenoidosa*.

**Fig. S3** GO functional classification of upregulated (A) and downregulated DEGs (B).

**Fig. S4** KEGG enrichment analysis of downregulated DEGs (A) and DAMs (B).

**Fig. S5** Heatmaps of DEGs related to photosynthesis (A) and antenna proteins (B).

**Fig. S6** Heatmap of DEGs related to antioxidation.

**Fig. S7** Relative abundance of DAMs associated with antioxidation in different treatment groups.

**Fig. S8** The relative abundance distribution of the top 12 ARG hosts at the phylum level in each group.

**Fig. S9** Genomic information of *Allorhizobium*_sp1 and *Methylobacteium*_sp1.

**Fig. S10** Annotation of KEGG pathways for strain *Pseudomonas*_sp1 genome.

**Fig. S11** Annotation of KEGG pathways for strain *Allorhizobium*_sp1 genome.

**Fig. S12** Annotation of KEGG pathways for strain *Methylobacteium*_sp1 genome.

**Fig. S13** Pathways of vitamin B6 metabolism.

**Fig. S14** UPLC-MS/MS chromatograms of FF, FF-D3, pyridoxal, and pyridoxal-D3.

**Table captions**

**Table S1.** The concentration of FF in the medium of each group in the pre-experiment.

**Table S2**. Mobile phase elution gradients.

**Table S3**. Grouping for co-culture experiment of phycospheric isolates and *A. pyrenoidosa*.

**Table S4**. UPLC-MS/MS conditions for FF, FF-D3, pyridoxal, and pyridoxal-D3 determination.

**Table S5.** The table of secondary metabolites synthesis gene clusters in *Pseudomonas*_sp1.

**Table S6.** The table of secondary metabolites synthesis gene clusters in *Methylobacterium*_sp1.

**Table S7.** The table of secondary metabolites synthesis gene clusters in *Allorhizobium*_sp1.

**Table S8.** The minimum inhibitory concentration (MIC) of three phycospheric bacteria isolates.

**Table S9.** The concentration of FF in the medium of each group in the co-culture experiment of *Pseudomonas*_sp1 and axenic *A. pyrenoidosa*.

**Table S10** Groups for pyridoxal addition experiments

**Text S1.** **Details of the pre-experiment**

In the pre-experiment, 1 mL of PLA MPs stock solution (2 g/L) and 200 μL of FF stock solution (0.1 g/L or 10 g/L) were added to the 200-mL medium cultured only with axenic *A. pyrenoidosa* and the medium co-cultured with axenic *A. pyrenoidosa* and bacteria. The experimental grouping and culture conditions were the same as those of the formal experiment. Previous studies showed that bacterial recruitment in the phycosphere of *Chlorella* sp. reached a plateau within 7 days [1]. Therefore, to explore the effect of FF on bacterial colonization, we focused on the degradation of FF in the medium in the first 7 days of incubation. On days 0, 3, and 7, 1-mL algal liquid was taken through a 0.45-μm filter membrane to determine the concentration of FF in the filtrate by ultra-performance liquid chromatography mass spectrometry (UPLC-MS/MS). The result showed no significant change in FF concentration in each treatment (Table S1). Therefore, we did not add additional FF to the co-culture system during the whole incubation period.

**Text S2.** **Determination of physiological properties of *A. pyrenoidosa***

**S2-1 Measurement of physiological indicators**

The optical density (OD) of microalgal solution was measured at a wavelength of 680 nm every 48 h and then transferred to algal cell numbers through standard curves to determine the growth of *A. pyrenoidosa* in each treatment. At days 6, 12, 18, 30, 42, and 60 of incubation, 10 mL of microalgal suspensions in each treatment were collected for quantification of photosynthetic pigments, extracellular polymeric substances (EPS), and antioxidant capacity. Photosynthetic pigments, including chlorophyll a (Chl-a), chlorophyll b (Chl-b), and carotenoids, were measured using 90% acetone freezing extraction [2]. EPS was extracted from *A. pyrenoidosa* using a thermal extraction method [3]. The extracellular protein content was measured by the assay kit, and the exopolysaccharide content was measured by the phenol-sulfuric acid colorimetry method [4]. The antioxidant capacity of *A. pyrenoidosa* was quantified by measuring the malondialdehyde (MDA) content and the activities of superoxide dismutase (SOD), catalase (CAT), and ATPase. They were assayed by the thiobarbituric acid method, hydroxylamine method, ammonium molybdate method, and molybdenum blue method in turn, respectively [5].

**S2-2 Morphological observation**

For morphological observation, the morphology of *A. pyrenoidosa* in each treatment was observed by scanning electron microscope (SEM) (S-4800, HITACHI, Japan) at the end of incubation. Briefly, *A. pyrenoidosa* cells were centrifugally collected, fixed with 2.5% glutaraldehyde solution at 4°C overnight, and rinsed three times with PBS. Subsequently, cells were dehydrated with an ascending series of ethanol (30%, 50%, 70%, 80%, 90%, and 100%), freeze-dried, and gold-sprayed for SEM observation.

**Text S3. Transcriptomic analysis**

**S3-1 RNA extraction**

Total RNA was extracted from *A. pyrenoidosa* cells using TRIzol® Reagent. Then RNA quality was determined by the 5300 Bioanalyser (Agilent) and quantified using the ND-2000 (NanoDrop Technologies). Only a high-quality RNA sample (OD260/280 = 1.8~2.2, OD260/230 ≥ 2.0, RQN ≥ 6.5) was used to construct the sequencing library.

**S3-2 Library preparation and sequencing**

The RNA-seq transcriptome library was prepared following Illumina® Stranded mRNA Prep, Ligation (San Diego, CA) using 1 μg of total RNA. Shortly, messenger RNA was isolated according to the polyA selection method by oligo (dT) beads, and then fragmented by the fragmentation buffer first. Secondly, double-stranded cDNA was synthesized using a SuperScript double-stranded cDNA synthesis kit (Invitrogen, CA) with random hexamer primers. Then the synthesized cDNA was subjected to end-repair, phosphorylation, and adapter addition according to the library construction protocol. Libraries were size-selected for cDNA target fragments of 300 bp on 2% Low Range Ultra Agarose, followed by PCR amplification using Phusion DNA polymerase (NEB) for 15 PCR cycles. After being quantified by Qubit 4.0, the sequencing library was performed on the NovaSeq X Plus platform (PE150) using the NovaSeq Reagent Kit.

**S3-3 Quality control and De novo Assembly**

The raw paired-end reads were trimmed and quality controlled by fastp[6] with default parameters. Then, clean data from the samples were used to do de novo assembly with Trinity [7]. To increase the assembly quality, all the assembled sequences were filtered by CD-HIT [8] and TransRate [9] and assessed with BUSCO (Benchmarking Universal Single-Copy Orthologs) [10]. The assembled transcripts were searched against the NCBI protein NR, Clusters of Orthologous Groups of proteins (COG), and Kyoto Encyclopedia of Genes and Genomes (KEGG) databases using Diamond to identify the proteins that had the highest sequence similarity with the given transcripts to retrieve their function annotations, and a typical cut-off E-value less than 1.0×10^−5^ was set. The BLAST2GO[11] program was used to get GO annotations of unique assembled transcripts for describing biological processes, molecular functions, and cellular components.

**S3-4 Differential expression analysis and functional enrichment**

To identify differentially expressed genes (DEGs) between two different groups, the expression level of each transcript was calculated according to the transcripts per million reads (TPM) method. RSEM[12] was used to quantify gene abundances. DEGs with |log2FC|≧1 and FDR < 0.05 were considered to be significantly different expressed genes. In addition, functional-enrichment analysis, including GO and KEGG, was performed to identify which DEGs were significantly enriched in GO terms and metabolic pathways at a Bonferroni-corrected P-value < 0.05 compared with the whole-transcriptome background. GO functional enrichment and KEGG pathway analysis were carried out by Goatools and Python scipy software, respectively.

**Text S4. Non-target metabolomic analysis**

**S4-1 Metabolite Extraction**

Metabolites were extracted from microalgal cells using a 400 µL methanol: water (4:1, v/v) solution with 0.02 mg/mL L-2-chlorophenylalanin as an internal standard. The mixture was allowed to settle at -10 °C and treated by High-throughput tissue crusher Wonbio-96c (Shanghai Wanbo Biotechnology Co., LTD) at 50 Hz for 6 min, then followed by ultrasound at 40 kHz for 30 min at 5 °C. The samples were placed at -20 °C for 30 min to precipitate proteins. After centrifugation at 13000 g at 4 °C for 15 min, the supernatant was carefully transferred to sample bottles for UHPLC-MS analysis.

**S4-2 Quality control sample**

As a part of the system conditioning and quality control process, a pooled quality control sample (QC) was prepared by mixing equal volumes of all samples. The QC samples were disposed of and tested in the same manner as the analytic samples. It helped to represent the whole sample set, which would be injected at regular intervals to monitor the stability of the analysis.

**S4-3 UHPLC-MS analysis**

The instrument platform for LC-MS analysis is the UHPLC-Q Exactive system of Thermo Fisher Scientific.

Chromatographic conditions:

2 μL of the sample was separated by an HSS T3 column (100 mm × 2.1 mm, 1.8 μm) and then entered into mass spectrometry detection. The mobile phases consisted of 0.1% formic acid in water: acetonitrile (95:5, v/v) (solvent A) and 0.1% formic acid in acetonitrile:isopropanol: water (47.5:47.5:5, v/v/v) (solvent B). The mobile phase elution gradients are shown in Table S2. The sample injection volume was 2 µL, and the flow rate was set to 0.4 mL/min. The column temperature was maintained at 40 °C. During the period of analysis, all these samples were stored at 4 °C.

MS conditions:

The mass spectrometric data were collected using a Thermo UHPLC-Q Exactive Mass Spectrometer equipped with an electrospray ionization (ESI) source operating in either positive or negative ion mode. The optimal conditions were set as follows: heater temperature, 400 °C; capillary temperature, 320 °C; sheath gas flow rate, 40 arb; aux gas flow rate, 10 arb; ion-spray voltage floating (ISVF), -2800 V in negative mode and 3500 V in positive mode, respectively; normalized collision energy, 20-40-60 V rolling for MS/MS. Full MS resolution was 70000, and MS/MS resolution was 17500. Data acquisition was performed with the Data Dependent Acquisition (DDA) mode. The detection was carried out over a mass range of 70-1050 m/z.

**S4-4 Data preprocessing and annotation**

After the mass spectrometry detection is completed, the raw data is preprocessed by Progenesis QI (Waters Corporation, Milford, USA) software, and a three-dimensional data matrix in CSV format is exported. The information in this three-dimensional matrix includes sample information, metabolite name, and mass spectral response intensity. Internal standard peaks, as well as any known false positive peaks (including noise, column bleed, and derivatized reagent peaks), were removed from the data matrix, deredundant, and peak-pooled. At the same time, the metabolites were searched and identified, and the main databases were the HMDB, Metlin, and Majorbio Database.

The data after the database search is uploaded to the Majorbio cloud platform (<https://cloud.majorbio.com>) for data analysis. Metabolic features detected at least 80% in any set of samples were retained. After filtering, minimum metabolite values were imputed for specific samples in which the metabolite levels fell below the lower limit of quantitation, and each metabolic feature was normalized by sum. To reduce the errors caused by sample preparation and instrument instability, the response intensity of the sample mass spectrum peaks was normalized by the sum normalization method, and the normalized data matrix was obtained. At the same time, variables with relative standard deviation (RSD) > 30% of QC samples were removed, and log10 logarithmization was performed to obtain the final data matrix for subsequent analysis.

**S4-5 Differential metabolites analysis**

Perform variance analysis on the matrix file after data preprocessing. The R package ropls (Version 1.6.2) performed principal component analysis (PCA) and orthogonal least partial squares discriminant analysis (OPLS-DA), and used 7-cycle interactive validation to evaluate the stability of the model. In addition, students' t-test and fold difference analysis were performed. The selection of significantly different metabolites was determined based on the variable importance in the projection (VIP) obtained by the OPLS-DA model and the *P*-value of the Student's t-test, and the metabolites with VIP > 1, *P* < 0.05 were significantly differentially accumulated metabolites (DAMs).

Differential metabolites among the two groups were summarized and mapped into their biochemical pathways through metabolic enrichment and pathway analysis based on database search (KEGG, http://www. genome.jp/kegg/). These metabolites can be classified according to the pathways they are involved or the functions they perform. Enrichment analysis was usually used to analyze a group of metabolites in a function node, whether they appear or not. The principle was that the annotation analysis of a single metabolite develops into an annotation analysis of a group of metabolites. The scipy.stats (Python packages) (https://docs.scipy.org/doc/scipy/) was exploited to identify statistically significantly enriched pathways using Fisher's exact test.

**Text S5.** **Metagenomic analysis**

**S5-1 Library construction and metagenomic sequencing**

DNA extract was fragmented to an average size of about 400 bp for paired-end library construction. Paired-end library was constructed using NEXTFLEX Rapid DNA-Seq (Bioo Scientific, USA). Paired-end sequencing was performed on Illumina NovaSeq (Illumina Inc., USA).

**S5-2 Sequence quality control and genome assembly**

The paired-end Illumina reads were trimmed of adaptors, and low-quality reads (length < 50 bp or with a quality value < 20 or having N bases) were removed by Fastp (version 0.20.0) [6]. The high-quality reads were then assembled using MEGAHIT (version 1.1.2) [13]. Contigs with a length ≥ 500 bp were selected as the final assembling result, and then the contigs were used for further gene prediction and annotation.

**S5-3 Gene prediction, taxonomy, and functional annotation**

Open reading frames (ORFs) from each assembled contig were predicted using Prodigal [14]. The predicted ORFs with a length ≥100 bp were retrieved and translated into amino acid sequences using the NCBI translation table. A non-redundant gene catalog was constructed using CD-HIT (version 4.7) [8] with 90% sequence identity and 90% coverage. High-quality reads were aligned to the non-redundant gene catalogs to calculate gene abundance with 95% identity using SOAPaligner (version 2.21) [15]. Representative sequences of the non-redundant gene catalog were aligned to the NR database for taxonomic annotations, the KEGG database for KEGG annotation, and the CARD database for ARGs annotation with an e-value cutoff of 1e-5 using Diamond (version 2.0.13) [16].

**Text S6. Calculation of ARGs abundance**

Gene abundance in each sample was performed using BWA's BWA-MEM algorithm based on coverage. The formula for calculating the abundance of ARG types or ARG subtypes belonging to ARG-like ORFs is as follows:

$$Abundance\left( coverage, \times/Gb \right)=\sum_{1}^{n} \frac{N_{mapped reads}\times L_{reads}/L_{ARG-like ORF(ARC)}}{S}$$

where $N_{mapped reads}$ is the number of clean reads matched by ARG-like ORFs or contigs carrying ARG (ARCs); $L_{reads}$ is the length of Illumina sequencing reads, 150 bp; $L_{ARG-like ORF(ARC)}$ is the sequence length of the target ARG-like ORFs or ARCs, bp; *n* is the number of ARG-like ORFs or ARCs belonging to the same classification; *S* is the size of the clean data dataset of the corresponding sample metagenome, Gb.

**Text S7. Identification of ARC hosts**

After extracting the amino acid sequences of ORFs in ARCs, the ORFs were annotated to the NCBI non-redundant (NR) with an e-value cutoff of 1e-5 using Diamond with the blastp method to identify the potential hosts of ARCs. The voting mechanism was subsequently used to determine the taxonomic annotation of ARCs' hosts: If more than 50% of the ARG-like ORFs on each ARC were assigned to the same taxon, the ARC was assigned to the corresponding taxonomic rank [17].

**Text S8. Identification of MGEs**

Based on the results of ARCs' annotation, the mobile genetic elements (MGEs) were determined according to whether the ORF matched one of the following keywords: transposase, transposon, intI1, integrase, integron, recombinase, and conjugative transfer protein [18].

**Text S9.** **Isolation of phycospheric bacteria**

Phycospheric bacteria were isolated using a dilution-to-extinction approach. Briefly, phycospheric bacteria were isolated by continuous centrifugation and sonication. For isolation, algal suspensions were centrifuged at 8000 r/min for 5 min to recover the supernatant. The pellet was washed with PBS buffer, followed by sonication for 5 min and centrifugation at 8000 r/min for 5 min. The supernatants from the first and second centrifugations were pooled together and diluted at 1:100, 1:1000, or 1:10000. The diluted supernatants were then spread on medium plates. After incubation for 72 h at 30 °C in the dark, visible bacteria growing on plates were picked for 16S rRNA amplicon sequencing.

**Text S10. Whole genome sequencing of** **phycospheric isolates**

**S10-1 Genomic DNA extraction**

The phycospheric isolate was streaked on LB solid medium and cultured at 30 ℃ for 12 h. Next, a single colony on the solid medium was inoculated into 200 mL of LB liquid medium and cultured at 30 ℃ for approximately 12 h at 150 rpm. The cell biomass was harvested after 10 min centrifugation at 12,000 × g. Genomic DNA of the phycospheric isolate was extracted using a Bacterial DNA extraction kit (magnetic beads) (Majorbio, Shanghai, China) according to the manufacturer’s protocol. Purified genomic DNA was quantified, and high-quality DNA was used to do further research.

**S10-2 Library construction and genome sequencing**

The genome was sequenced using a combination of Illumina sequencing platforms and Nanopore PromethION sequencing platforms (MajorBio Co., Shanghai, China).

For Illumina sequencing, genomic DNA was used for each strain in sequencing library construction. DNA samples were sheared into 400-500 bp fragments using a Covaris M220 Focused Acoustic Shearer following the manufacturer’s protocol. Illumina sequencing libraries were prepared from the sheared fragments using the NEXTFLEX Rapid DNA-Seq Kit. Briefly, 5’ ends were first end-repaired and phosphorylated. Next, the 3’ ends were A-tailed and ligated to sequencing adapters. The third step is to enrich the adapter-ligated products using PCR. The prepared libraries were then used for paired-end Illumina sequencing (2 × 150 bp) on an Illumina Novaseq 6000 (Illumina Inc., San Diego, CA, USA).

For Nanopore sequencing, DNA fragments were repaired, purified, and then attached sequencing adapters supplied in the SQK-LSK109 kit to the DNA ends. Next, the Nanopore library was prepared and sequenced on Oxford Nanopore PromethION (Oxford Nanopore, Oxford, UK).

**S10-3 Genome assembly and annotation**

The raw Illumina sequencing reads generated from the paired-end library were subjected to quality filtering using fastp v0.23.0. Nanopore reads were extracted, basecalled, demultiplexed, and trimmed with the minimum Q score cutoff of 7. Then the clean short and long reads were assembled to construct complete genomes using Unicycle v0.4.8 [19]. As a final step, Unicycler uses Pilon v1.22 to polish the assembly using short-read alignments, reducing the rate of small errors. The coding sequences (CDs) of the chromosome and plasmid were predicted using Glimmer [20] and GeneMarkS [21], respectively. tRNA-scan-SE (v 2.0)[22] was used for tRNA prediction and Barrnap v0.9 (https://github.com/tseemann/barrnap) was used for rRNA prediction. The predicted CDs were annotated from NR, Swiss-Prot, Pfam, GO, COG, and KEGG databases using sequence alignment tools such as BLASTP, Diamond, and HMMER. Briefly, each set of query proteins was aligned with the databases, and annotations of best-matched subjects (e-value < 10^-5^) were obtained for gene annotation. Biosynthetic gene clusters (BGCs) of secondary metabolites were identified by antiSMASH v5.1.2 software.

**Text S11. Determination of the** **minimum inhibitory concentration (MIC) of phycospheric isolates on FF**

The minimum inhibitory concentrations (MIC) of different phycospheric isolates on FF were determined by a method of 96-well microtiter assay. Specifically, the FF mother liquor was diluted in multiples to give a final concentration range of 4 μg/mL to 256 μg/mL. Then, 10 μL of FF solution, 10 μL of bacterial solution (10^6^ CFU/mL), and 80 μL of MH medium were added to each well of a sterile 96-well plate and incubated at 30℃ for 16-18 h. The wells without bacterial solution and without FF solution were used as negative and positive controls, respectively. Three biological replicates were performed for each of the above treatments. The criterion of MIC was that the OD_600_ value of the tested sample was the same as that of the negative control.

**Text S12. Co-culture of phycospheric isolates and *A. pyrenoidosa***

In the co-culture experiment, the selected phycospheric bacteria and axenic *A. pyrenoidosa* were co-inoculated into conical flasks containing 400 mL BG-11 medium at an initial density of 1.0 × 10^5^ ind/mL for *A. pyrenoidosa* and 1.0 × 10^7^ CFU/mL for bacteria. To further test whether phycospheric bacteria could promote the adaptation of *A. pyrenoidosa* to FF and PLA MPs exposure, FF and PLA MPs were added to the co-culture system. Five different treatments were set up, as shown in Table S3. Each treatment was performed in triplicate, and these flasks were incubated for 14 days at 25 ± 1°C, illuminated with 3000 ± 300 lux with a 12h/12h light/dark interval, and shaken at least three times daily.

**Text S13. Quantification of FF and the metabolite pyridoxal**

The concentration of FF and pyridoxal in the medium was determined by ultra-performance liquid chromatography mass spectrometry (UPLC-MS/MS) (Waters ACQUITY UPLC H-Class coupled to Xevo TQ).

For the determination of FF, analytes were separated using an ACQUITY UPLC BEH C18 column (2.1 × 100 mm, 1.7 μm). The mobile phase, consisting of ultrapure water (A) and methanol (B), was used in gradient elution as follows: (Tmin/methanol): 0.0–1.0/20%, 1.0–4.0/90%, 4.0-5.0/90%, 5.0-5.1/20%, and 5.1–7.0/20%. The injection volume was 5 μL, and the flow rate was set at 0.25 mL/min. The column temperature was maintained at 40°C. The mass spectrometric data were collected using a mass spectrometer (Xevo TQ) equipped with an electrospray ionization (ESI) source operating in negative ion mode. The optimal conditions were set as follows: capillary voltage at 2500 V; desolvation temperature at 350℃; desolvation gas flow at 650 L/Hr; collision gas flow at 0.15 mL/min. FF and FF-D3 were detected in multiple reaction monitoring (MRM) mode as shown in Table S4. The mass spectra of FF and FF-D3 are shown in Fig. S14A.

For the determination of pyridoxal, analytes were separated using an ACQUITY UPLC BEH C18 column (2.1 × 50 mm, 1.7 μm). The mobile phase, consisting of 0.1% formic acid in ultrapure water (A) and 0.1% formic acid in methanol (B), was used in gradient elution as follows: (Tmin/B): 0.0–2.0/5%, 2.0–4.0/80%, 4.0-4.1/5%, and 4.1–7.0/5%. The injection volume was 2 μL, and the flow rate was set at 0.2 mL/min. The column temperature was maintained at 40°C. The mass spectrometric data were collected using a mass spectrometer (Xevo TQ) equipped with an electrospray ionization (ESI) source operating in positive ion mode. The optimal conditions were set as follows: capillary voltage at 2500 V; desolvation temperature at 350℃; desolvation gas flow at 650 L/Hr; collision gas flow at 0.15 mL/min. Pyridoxal and pyridoxal-D3 were detected in multiple reaction monitoring (MRM) mode as shown in Table S4. The mass spectra of pyridoxal and pyridoxal-D3 are shown in Fig. S14B.

**Text S14. Co-culture of *Pseudomonas_*sp1, pyridoxal, and *A. pyrenoidosa***

In the co-culture experiment, *Pseudomonas_*sp1 and axenic *A. pyrenoidosa* were co-inoculated into conical flasks containing 400 mL BG-11 medium at an initial density of 1.0 × 10^5^ ind/mL for *A. pyrenoidosa* and 1.0 × 10^7^ CFU/mL for bacteria. To further test whether pyridoxal could promote the adaptation of *A. pyrenoidosa* to FF exposure, FF and pyridoxal were added to the co-culture system. The treatment concentrations of pyridoxal were selected according to the detected concentration of pyridoxal in the aforementioned experiment. Eight different treatments were set up, as shown in Table S10. Each treatment was performed in triplicate, and these flasks were incubated for 30 days at 25 ± 1°C, illuminated with 3000 ± 300 lux with a 12h/12h light/dark interval, and shaken at least three times daily.

**References**

1. Krespach MKC, Stroe MC, Flak M, Komor AJ, Nietzsche S, Sasso S, Hertweck C, Brakhage AA: **Bacterial marginolactones trigger formation of algal gloeocapsoids, protective aggregates on the verge of multicellularity**. *Proceedings of the National Academy of Sciences* 2021, **118**(45).

2. Jia J, Liu Q, Wu C: **Microplastic and antibiotic proliferated the colonization of specific bacteria and antibiotic resistance genes in the phycosphere of Chlorella pyrenoidosa**. *J Hazard Mater* 2023, **455**:131618-131628.

3. Yan Z, Xu L, Zhang W, Yang G, Zhao Z, Wang Y, Li X: **Comparative toxic effects of microplastics and nanoplastics on Chlamydomonas reinhardtii: Growth inhibition, oxidative stress, and cell morphology**. *Journal of Water Process Engineering* 2021, **43**:102291-102301.

4. Tan B, Hu P, Niu X, Zhang X, Liu J, Frenken T, Hamilton PB, Haffner GD, Chaganti SR, Nwankwegu AS *et al*: **Microbial community day-to-day dynamics during a spring algal bloom event in a tributary of Three Gorges Reservoir**. *Sci Total Environ* 2022, **839**:156183-156195.

5. Yusefi-Tanha E, Fallah S, Rostamnejadi A, Pokhrel LR: **Zinc oxide nanoparticles (ZnONPs) as a novel nanofertilizer: Influence on seed yield and antioxidant defense system in soil grown soybean (Glycine max cv. Kowsar)**. *Sci Total Environ* 2020, **738**:140240-140252.

6. Chen S, Zhou Y, Chen Y, Gu J: **fastp: an ultra-fast all-in-one FASTQ preprocessor**. *Bioinformatics* 2018, **34**(17):i884-i890.

7. Grabherr MG, Haas BJ, Yassour M, Levin JZ, Thompson DA, Amit I, Adiconis X, Fan L, Raychowdhury R, Zeng Q *et al*: **Full-length transcriptome assembly from RNA-Seq data without a reference genome**. *Nat Biotechnol* 2011, **29**(7):644-652.

8. Fu L, Niu B, Zhu Z, Wu S, Li W: **CD-HIT: accelerated for clustering the next-generation sequencing data**. *Bioinformatics* 2012, **28**(23):3150-3152.

9. Smith-Unna R, Boursnell C, Patro R, Hibberd JM, Kelly S: **TransRate: reference-free quality assessment of de novo transcriptome assemblies**. *Genome Res* 2016, **26**(8):1134-1144.

10. Manni M, Berkeley MR, Seppey M, Simão FA, Zdobnov EM, Kelley J: **BUSCO update: Novel and streamlined workflows along with broader and deeper phylogenetic coverage for scoring of eukaryotic, prokaryotic, and viral genomes**. *Molecular Biology and Evolution* 2021, **38**(10):4647-4654.

11. Conesa A, Götz S, García-Gómez JM, Terol J, Talón M, Robles M: **Blast2GO: a universal tool for annotation, visualization and analysis in functional genomics research**. *Bioinformatics* 2005, **21**(18):3674-3676.

12. Li B, Dewey CN: **RSEM: accurate transcript quantification from RNA-Seq data with or without a reference genome**. *BMC Bioinformatics* 2011, **12**(1).

13. Li D, Liu C-M, Luo R, Sadakane K, Lam T-W: **MEGAHIT: an ultra-fast single-node solution for large and complex metagenomics assembly via succinct de Bruijn graph**. *Bioinformatics* 2015, **31**(10):1674-1676.

14. Hyatt D, Chen G-L, LoCascio PF, Land ML, Larimer FW, Hauser LJ: **Prodigal: prokaryotic gene recognition and translation initiation site identification**. *BMC Bioinformatics* 2010, **11**(1).

15. Li R, Li Y, Kristiansen K, Wang J: **SOAP: short oligonucleotide alignment program**. *Bioinformatics* 2008, **24**(5):713-714.

16. Buchfink B, Xie C, Huson DH: **Fast and sensitive protein alignment using DIAMOND**. *Nature Methods* 2014, **12**(1):59-60.

17. Ma L, Li B, Jiang X-T, Wang Y-L, Xia Y, Li A-D, Zhang T: **Catalogue of antibiotic resistome and host-tracking in drinking water deciphered by a large scale survey**. *Microbiome* 2017, **5**(1):154.

18. Forsberg KJ, Patel S, Gibson MK, Lauber CL, Knight R, Fierer N, Dantas G: **Bacterial phylogeny structures soil resistomes across habitats**. *Nature* 2014, **509**(7502):612-616.

19. Phillippy AM, Wick RR, Judd LM, Gorrie CL, Holt KE: **Unicycler: Resolving bacterial genome assemblies from short and long sequencing reads**. *PLOS Computational Biology* 2017, **13**(6).

20. Delcher A, Bratke K, Powers E, Salzberg S: **Identifying bacterial genes and endosymbiont DNA with Glimmer**. *Bioinformatics* 2007, **23**(6):673-679.

21. Besemer J, Borodovsky M: **GeneMark: web software for gene finding in prokaryotes, eukaryotes and viruses**. *Nucleic Acids Research* 2005, **33**(Web Server):W451-W454.

22. Chan PP, Lowe TM: **tRNAscan-SE: Searching for tRNA genes in genomic sequences**. In: *Gene Prediction.* 2019: 1-14.


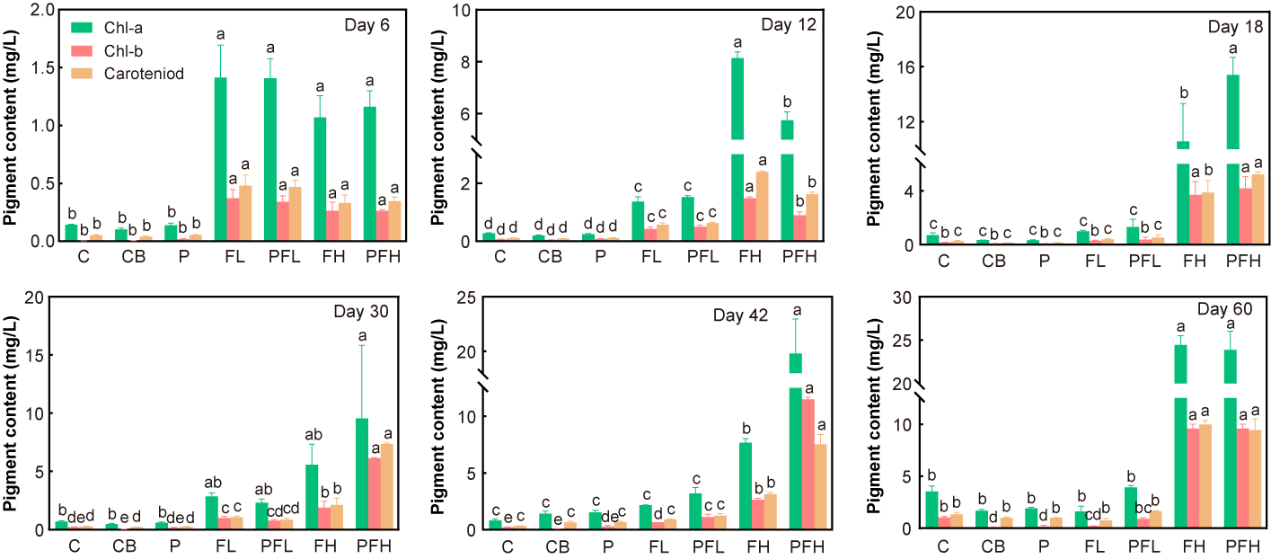


**Fig. S1** **Changes in photosynthetic pigment content of *A. pyrenoidosa* under PLA MPs and FF treatment.** Different letters indicate significant differences between different groups (*P* < 0.05, one-way ANOVA).


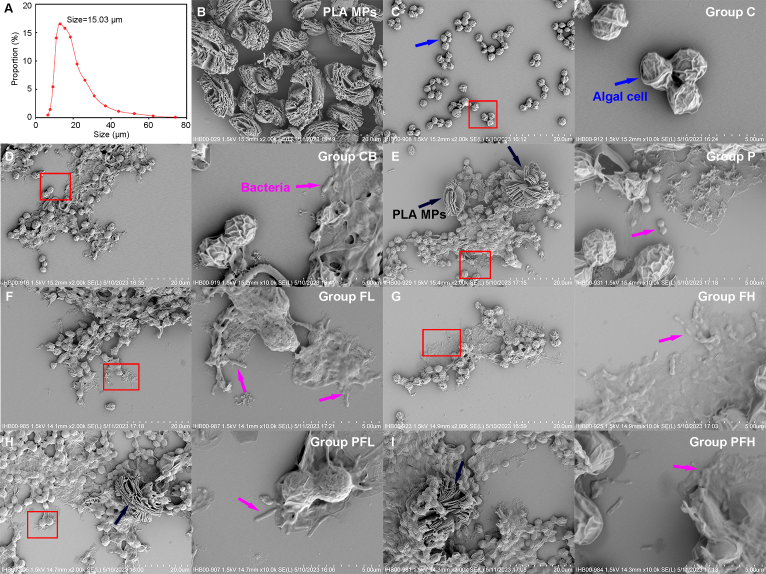


**Fig. S2 Morphological characteristics of PLA MPs and *A. pyrenoidosa*.** (A, B) The particle size distribution (A) and SEM image (B) of PLA MPs. (C-I) The SEM images of different treatment groups. The red box indicates the enlarged area. The blue, purple, and black arrows indicate *A. pyrenoidosa*, bacteria, and PLA MPs in the phycosphere, respectively.

**
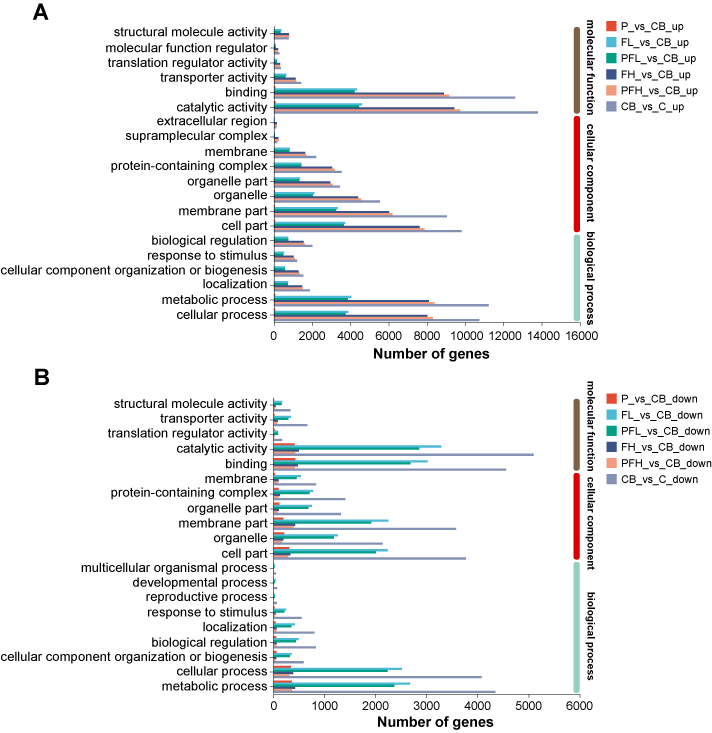
**

**Fig. S3 GO functional classification of upregulated (A) and downregulated (B) DEGs.**

**
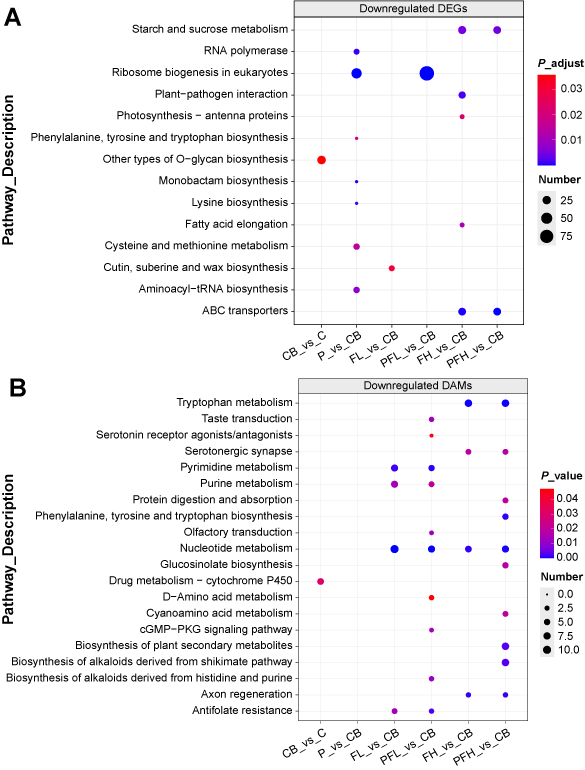
**

**Fig. S4 KEGG enrichment analysis of downregulated DEGs (A) and DAMs (B).**

**
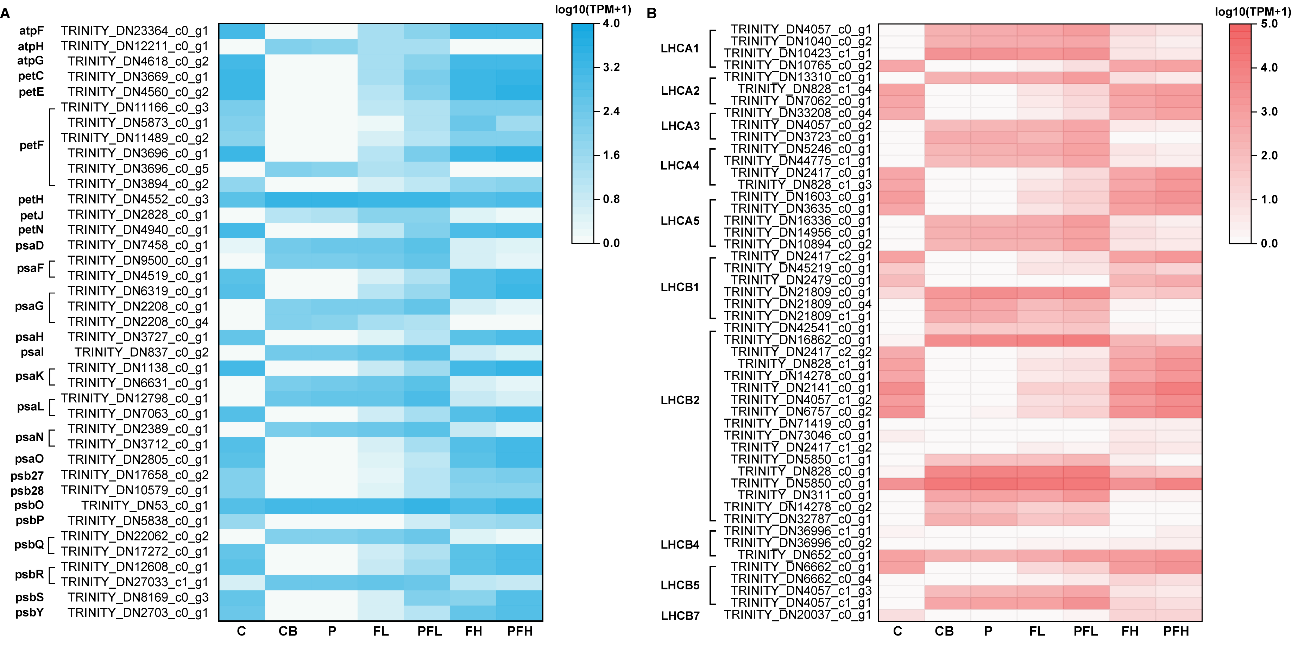
**

**Fig. S5 Heatmaps of DEGs related to photosynthesis (A) and antenna proteins (B).**

**
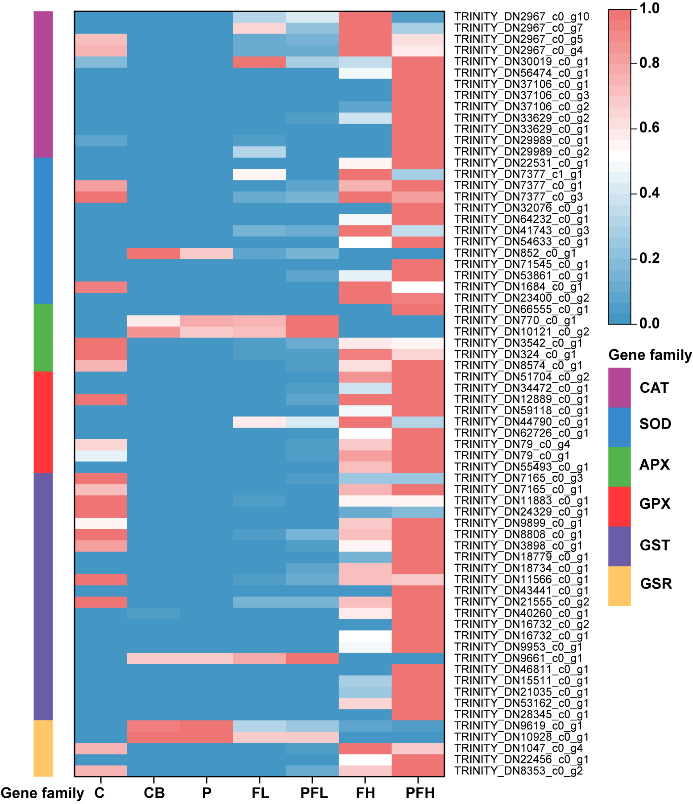
**

**Fig. S6 Heatmap of DEGs related to antioxidation.**

**
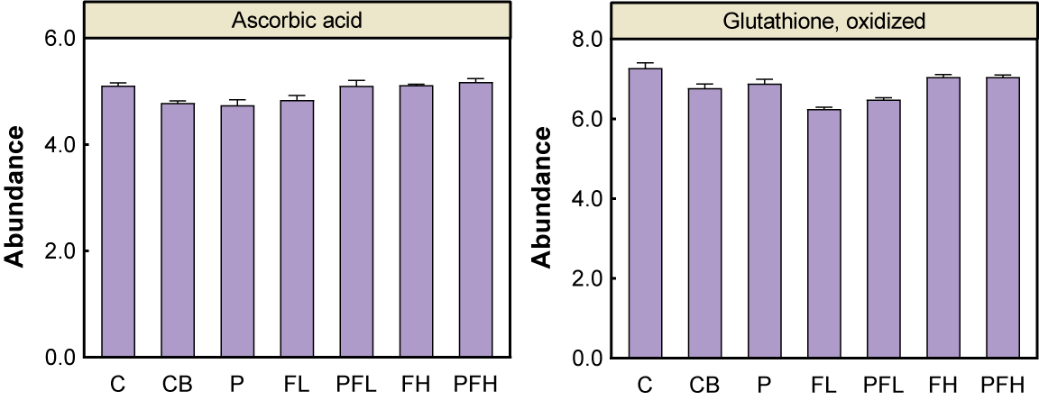
**

**Fig. S7 Relative abundance of DAMs associated with antioxidation in different treatment groups.**

**
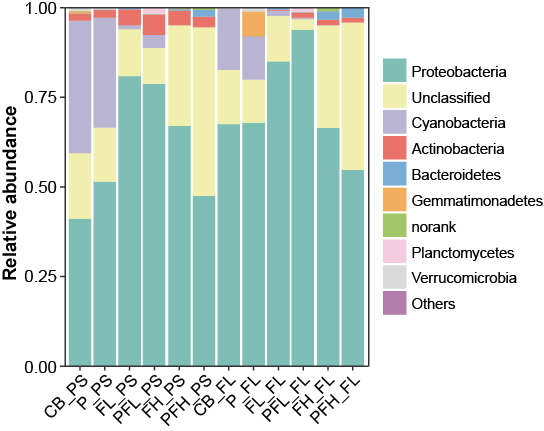
**

**Fig. S8** The relative abundance distribution of the top 12 ARG hosts at the phylum level in each group.

**
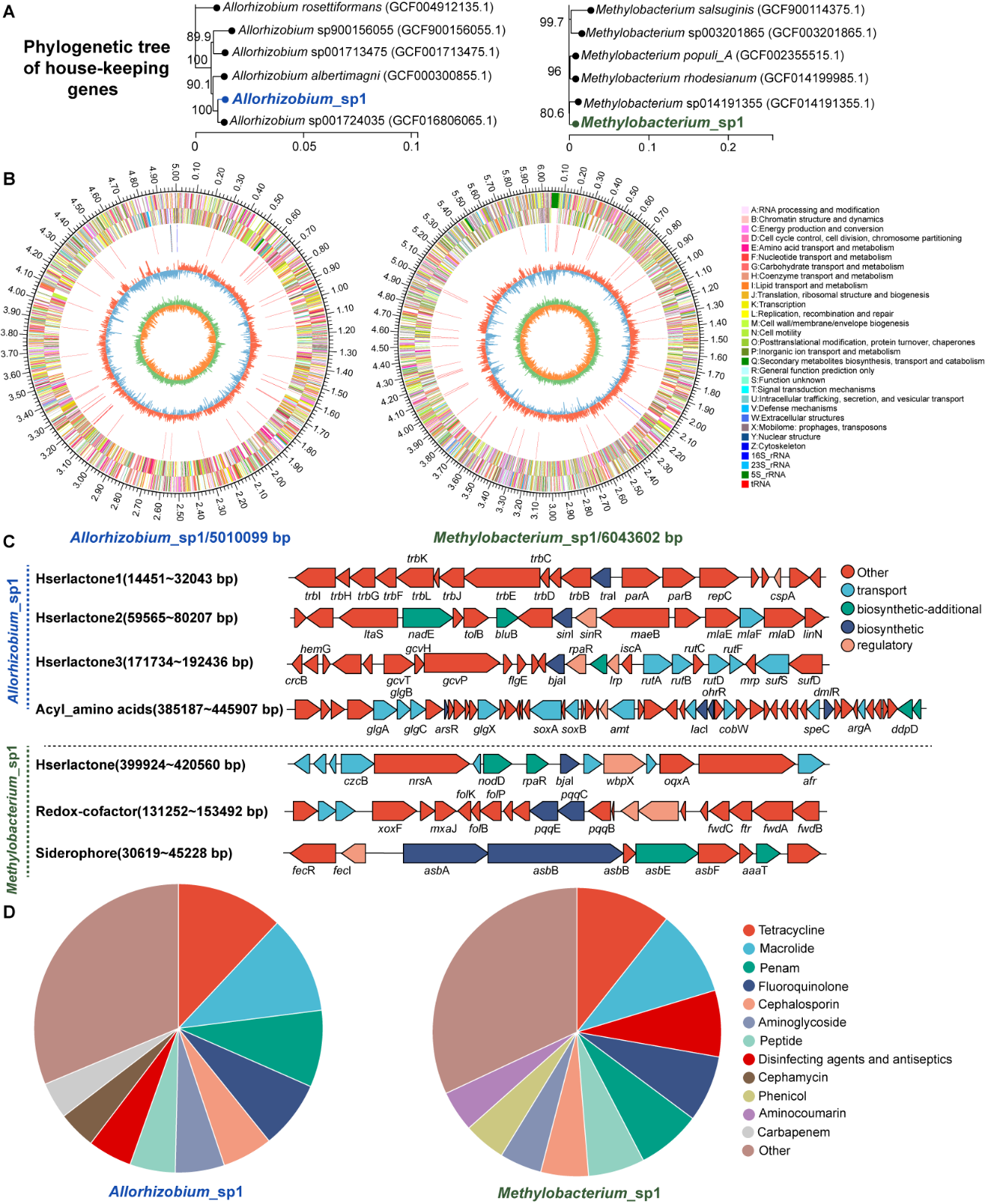
**

**Fig. S9 Genomic information of *Allorhizobium*_sp1 and *Methylobacteium*_sp1.** (A) Phylogenetic tree of *Allorhizobium*_sp1 and *Methylobacteium*_sp1 based on house-keeping genes. Numbers listed at the branches were bootstrap values. (B) Genetic map of *Allorhizobium*_sp1 and *Methylobacteium*_sp1. From the outside to the inner: Circle 1, the genome size. Circle 2 and 3, protein coding regions by COG function categories on forward/reverse strand, different categories of COG pathways were showed using different colors. Circle 4, the distribution of rRNA and tRNA. Circle 5, the GC content, the outer red part indicates that the GC content of this region is higher than the average GC content of the whole genome, and the inner blue part is the opposite. Circle 6, the GC-Skew value (G-C/G+C). (C) Linear map of predicted secondary metabolite synthesis gene clusters. (D) Prediction of antibiotic resistance gene types.

**
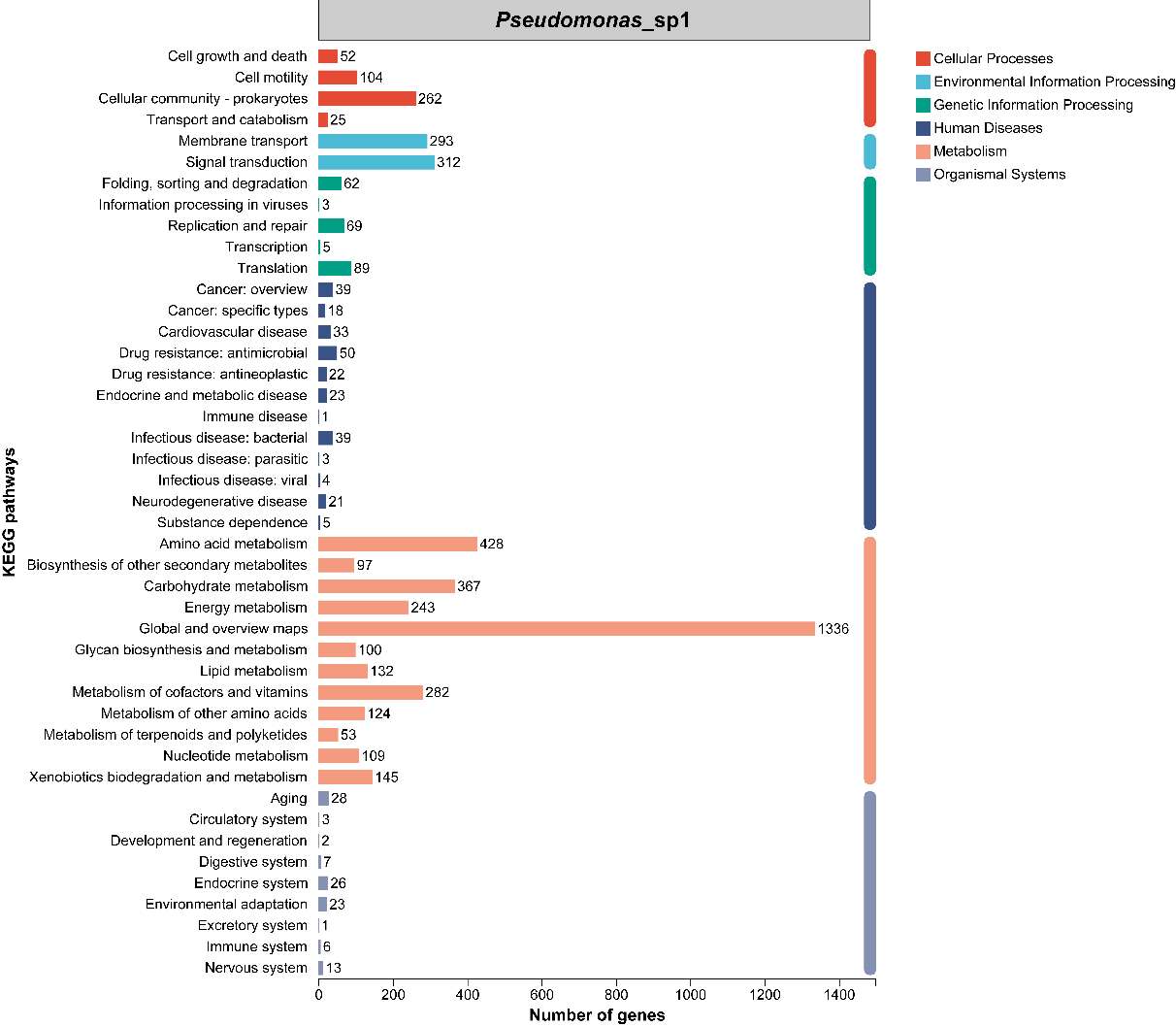
**

**Fig. S10 Annotation of KEGG pathways for strain *Pseudomonas*_sp1 genome.**

**
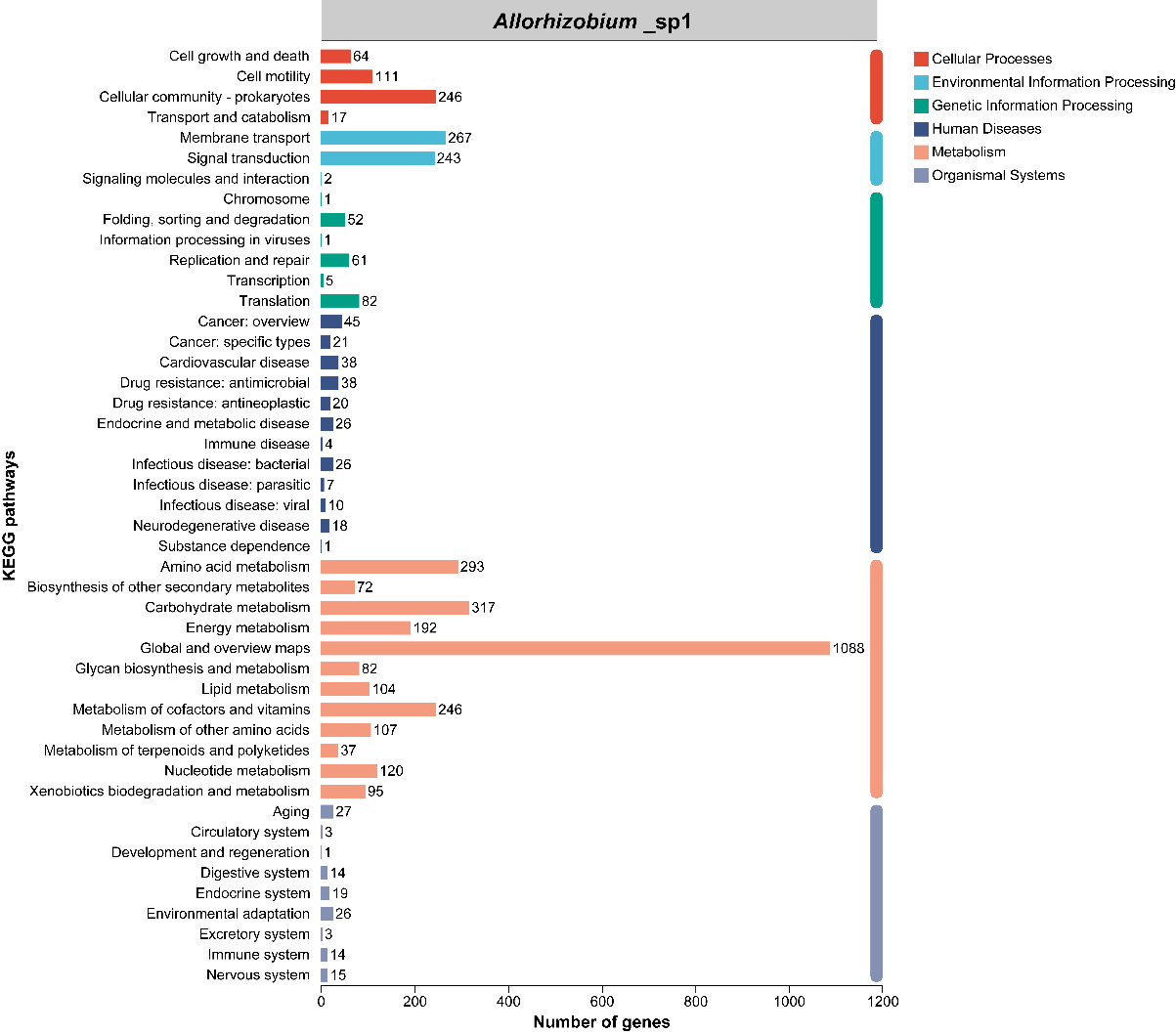
**

**Fig. S11 Annotation of KEGG pathways for strain *Allorhizobium*_sp1 genome.**

**
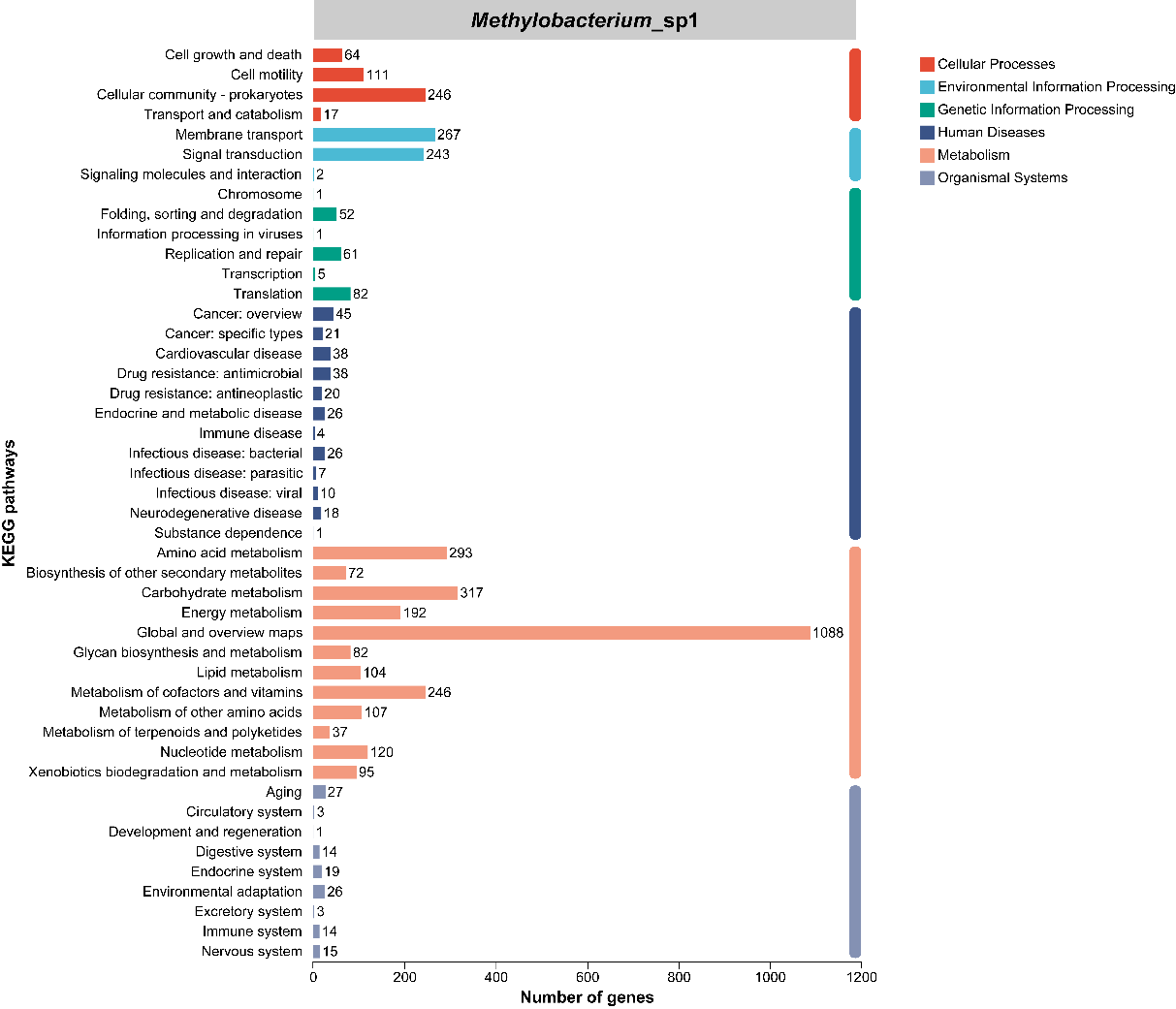
**

**Fig. S12 Annotation of KEGG pathways for strain *Methylobacteium*_sp1 genome.**

**
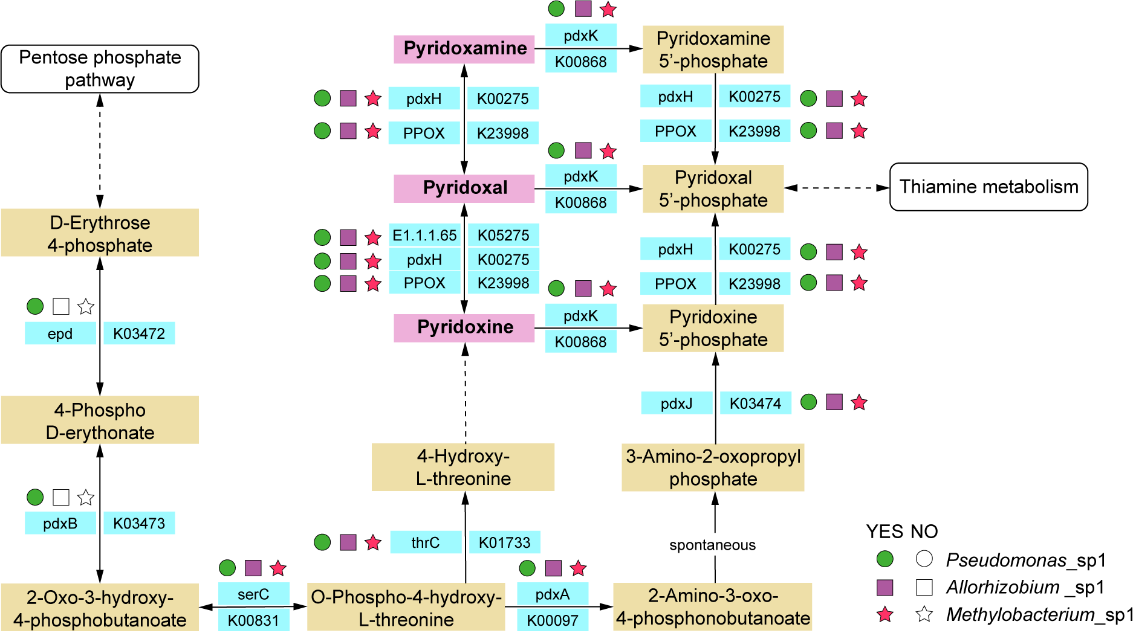
**

**Fig. S13** **Pathways of vitamin B6 metabolism.**

**
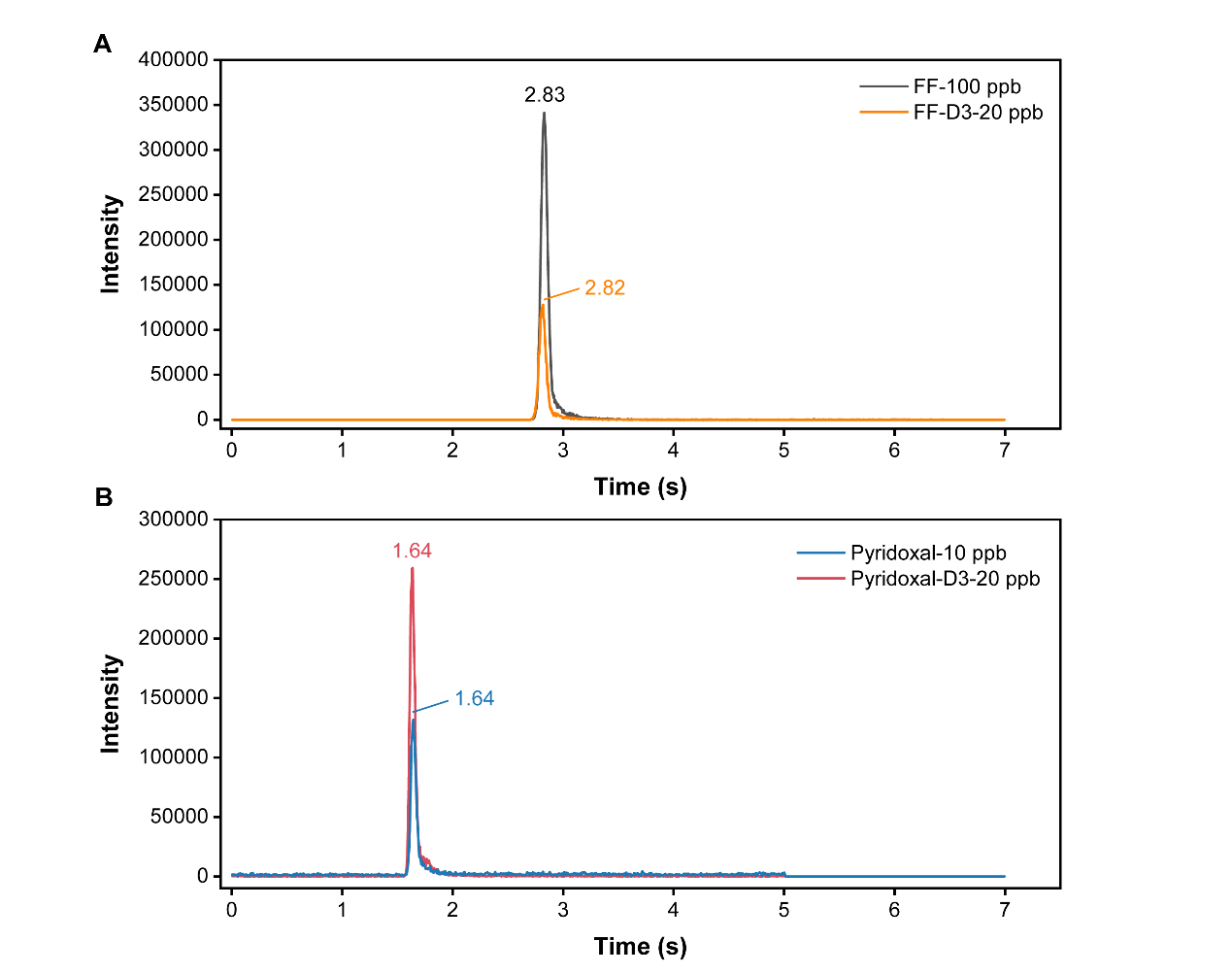
**

**Fig. S14 UPLC-MS/MS chromatograms of FF, FF-D3, pyridoxal, and pyridoxal-D3.**

**Table S1.** The concentration of FF in the medium of each group in the pre-experiment. (Unit: mg/L)

|  | Axenic *A. pyrenoidosa* | | *A. pyrenoidosa* with bacteria | |
| --- | --- | --- | --- | --- |
| Group | Day 3 | Day 7 | Day 3 | Day 7 |
| FL | 0.089 | 0.084 | 0.094 | 0.087 |
| PFL | 0.097 | 0.098 | 0.099 | 0.093 |
| FH | 9.626 | 9.856 | 9.494 | 9.476 |
| PFH | 9.953 | 9.812 | 9.488 | 9.982 |

Notes: FL: 0.1 mg/L FF; PFL: 0.1 mg/L FF + 10 mg/L PLA MPs; FH: 10 mg/L FF; PFH: 10 mg/L FF + 10 mg/L PLA MPs.

**Table S2.** Mobile phase elution gradients.

| Time (min) | Solvent A (%) | Solvent B (%) |
| --- | --- | --- |
| 0 | 100 | 0 |
| 0.1 | 95 | 5 |
| 2 | 75 | 25 |
| 9 | 0 | 100 |
| 13 | 0 | 100 |
| 13.1 | 100 | 0 |
| 16 | 100 | 0 |

**Table S3.** Grouping for co-culture experiment of phycospheric isolates and *A. pyrenoidosa*.

| Group | Axenic *A. pyrenoidosa* | Phycospheric bacteria | FF (mg/L) | PLA MPs (mg/L) |
| --- | --- | --- | --- | --- |
| C | + | - | 0 | 0 |
| CB | + | + | 0 | 0 |
| CF | + | - | 10 | 0 |
| CBF | + | + | 10 | 0 |
| CBFP | + | + | 10 | 10 |

**Table S4.** UPLC-MS/MS conditions for FF, FF-D3, pyridoxal, and pyridoxal-D3 determination.

| Compound | Ion mode | Parent ion  (m/z) | Daughter ion  (m/ z) | Dwell  (s) | Cone (V) | Collision (V) |
| --- | --- | --- | --- | --- | --- | --- |
| FF | Negative | 356.0 | 185.0 | 0.106 | 20 | 20 |
| FF | Negative | 356.0 | 336.0 | 0.106 | 20 | 10 |
| FF-D3 | Negative | 359.0 | 188.0 | 0.106 | 20 | 20 |
| Pyridoxal | Positive | 168.0 | 94.0 | 0.078 | 20 | 20 |
| Pyridoxal | Positive | 168.0 | 150.0 | 0.078 | 20 | 15 |
| Pyridoxal-D3 | Positive | 171.1 | 97.1 | 0.078 | 20 | 25 |
| Pyridoxal-D3 | Positive | 171.1 | 153.1 | 0.078 | 20 | 20 |

**Table S5.** The table of secondary metabolites synthesis gene clusters in *Pseudomonas*_sp1.

| Bacteria | Type | Start | End | MIBiG accession | Similar Cluster | Similarity (%) | Gene No. |
| --- | --- | --- | --- | --- | --- | --- | --- |
| *Pseudomonas*_sp1 | redox-cofactor | 404949 | 427109 | BGC0001100 | lankacidin C | 13 | 16 |
|  | RiPP-like | 1152027 | 1162307 | - | - | - | 10 |
|  | NRPS | 1773714 | 1825799 | BGC0000413 | pyoverdin | 7 | 38 |
|  | NRPS | 1840845 | 1921433 | BGC0000413 | pyoverdin | 10 | 40 |
|  | RRE-containing | 2552370 | 2572438 | - | - | - | 17 |
|  | ranthipeptide | 3629053 | 3650484 | BGC0000413 | pyoverdin | 9 | 21 |
|  | RiPP-like | 4247160 | 4257994 | - | - | - | 9 |
|  | T1PKS | 4851143 | 4898643 | BGC0000773 | lipopolysaccharide | 25 | 33 |
|  | NAGGN | 4926155 | 4941047 | - | - | - | 12 |
|  | thiopeptide | 5730048 | 5754634 | - | - | - | 25 |

**Table S6.** The table of secondary metabolites synthesis gene clusters in *Methylobacterium*_sp1.

| Bacteria | Type | Start | End | MIBiG accession | Similar Cluster | Similarity (%) | Gene No. |
| --- | --- | --- | --- | --- | --- | --- | --- |
| *Methylobacterium*_sp1 | terpene | 653153 | 674227 | BGC0000647 | carotenoid | 100 | 21 |
|  | NRPS | 244799 | 288336 | - | - | - | 33 |
|  | hserlactone | 399924 | 420560 | - | - | - | 16 |
|  | terpene | 70339 | 91236 | - | - | - | 20 |
|  | NAPAA | 376671 | 410871 | - | - | - | 32 |
|  | T1PKS | 227368 | 264748 | - | - | - | 28 |
|  | redox-cofactor | 131252 | 153492 | - | - | - | 23 |
|  | siderophore | 30619 | 45228 | - | - | - | 10 |

**Table S7.** The table of secondary metabolites synthesis gene clusters in *Allorhizobium*_sp1.

| Bacteria | Type | Start | End | MIBiG accession | Similar Cluster | Similarity (%) | Gene No. |
| --- | --- | --- | --- | --- | --- | --- | --- |
| *Allorhizobium*_sp1 | NAGGN | 891462 | 910279 | - | - | - | 16 |
|  | T1PKS | 1 | 47662 | - | - | - | 34 |
|  | RiPP-like | 367578 | 378499 | - | - | - | 8 |
|  | acyl_amino_acids | 385187 | 445907 | - | - | - | 55 |
|  | terpene | 152816 | 173986 | - | - | - | 17 |
|  | hserlactone | 171734 | 192436 | - | - | - | 25 |
|  | terpene | 193585 | 214440 | - | - | - | 20 |
|  | hserlactone | 59565 | 80207 | - | - | - | 16 |
|  | hserlactone | 14302 | 32903 | - | - | - | 18 |
|  | hserlactone | 14451 | 32043 | - | - | - | 20 |

**Table S8.** The minimum inhibitory concentration (MIC) of three phycospheric bacteria isolates. (Unit: mg/L)

| Bacteria | *Pseudomonas*_sp1 | *Allorhizobium*_sp1 | *Methylobacteium*_sp1 |
| --- | --- | --- | --- |
| MIC | >256 | 16 | 8 |

**Table S9.** The concentration of FF in the medium of each group in the co-culture experiment of *Pseudomonas*_sp1 and axenic *A. pyrenoidosa*. (means ± SD, unit: mg/L)

| Group | Day 1 | Day 13 |
| --- | --- | --- |
| CF | 9.21±0.23 | 9.67±0.49 |
| CBF | 9.03±0.18 | 10.01±0.64 |
| CBFP | 9.26±0.11 | 10.02±0.13 |

**Table S10** Groups for pyridoxal addition experiments

| Group | Axenic *A. pyrenoidosa* | *Pseudomonas*_sp1 | FF (mg/L) | Pyridoxal (μg/L) |
| --- | --- | --- | --- | --- |
| C | + | -- | 0 | 0 |
| CF | + | -- | 10 | 0 |
| CF-5VB6 | + | -- | 10 | 5 |
| CF-10VB6 | + | -- | 10 | 10 |
| CB | + | + | 0 | 0 |
| CBF | + | + | 10 | 0 |
| CBF-5VB6 | + | + | 10 | 5 |
| CBF-10VB6 | + | + | 10 | 10 |
